# Transcriptional profiling at the *DLK1/MEG3* domain explains clinical overlap between imprinting disorders

**DOI:** 10.1126/sciadv.aau9425

**Published:** 2019-02-20

**Authors:** Walid Abi Habib, Frédéric Brioude, Salah Azzi, Sylvie Rossignol, Agnès Linglart, Marie-Laure Sobrier, Éloïse Giabicani, Virginie Steunou, Madeleine D. Harbison, Yves Le Bouc, Irène Netchine

**Affiliations:** 1 Sorbonne Université, INSERM, UMRS 938, Centre de Recherche Saint-Antoine, Paris, France.; 2 AP-HP, Hôpital Trousseau, Service d’Explorations Fonctionnelles Endocriniennes, Paris, France.; 3 Service de Génétique Médicale, Centre de Référence pour les Anomalies du Développement (FECLAD), Hôpitaux Universitaires de Strasbourg, Strasbourg, France.; 4 Endocrinology and Diabetology for Children and Reference Center for Rare Disorders of Calcium and Phosphate Metabolism, Bicêtre Paris Sud, AP-HP, Le Kremlin-Bicêtre, France.; 5 INSERM U986, INSERM, Le Kremlin-Bicêtre, France.; 6 Department of Pediatrics, Icahn School of Medicine at Mount Sinai, New York, NY, USA.

## Abstract

Imprinting disorders (IDs) often affect growth in humans, leading to diseases with overlapping features, regardless of the genomic region affected. IDs related to hypomethylation of the human 14q32.2 region and its *DLK1/MEG3* domain are associated with Temple syndrome (TS14). TS14 is a rare type of growth retardation, the clinical signs of which overlap considerably with those of Silver-Russell syndrome (SRS), another ID related to *IGF2* down-regulation at 11p15.5 region. We show that 14q32.2 hypomethylation affects expression, not only for genes at this locus but also for other imprinted genes, and especially lowers *IGF2* levels at 11p15.5. Furthermore, expression of nonimprinted genes is also affected, some of which are also deregulated in SRS patients. These findings highlight the epigenetic regulation of gene expression at the *DLK1/MEG3* domain. Expression profiling of TS14 and SRS patients highlights common signatures, which may account for the clinical overlap observed between TS14 and SRS.

## Teaser

Temple or Silver-Russell syndrome? We reveal the molecular reasons for the clinical resemblance between these two syndromes.

## INTRODUCTION

**Fig. 1:**
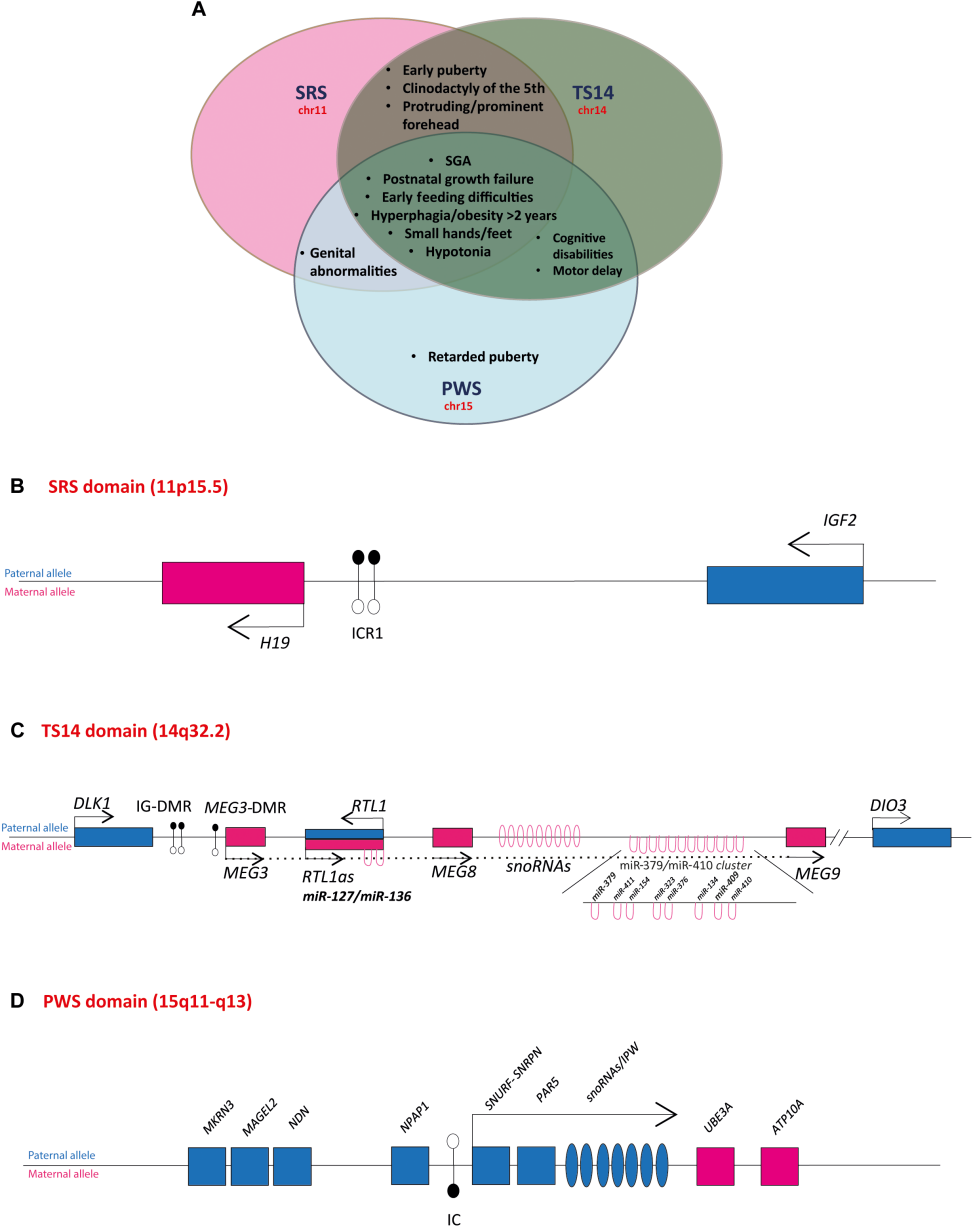
Overlap of clinical features between SRS, TS14, and PWS and DMRs with parent-specific gene expression from the *DLK1/MEG3, IGF2/H19*, and *SNURF/IPW* domains. (**A**) Schematic representation of the overlapping clinical features in SRS, TS14, and PWS patients. Schematic diagram of the regions imprinted in humans (**B**) the *IGF2/H19* domain on 11p15.5 and (**C**) the *DLK1/MEG3* domain of the 14q32.2 region. The relative positions of hairpin-like [pre-microRNA (miRNA)] structures within the miR-379/miR-410 cluster are indicated in the enlargement in the inset and (**D**) the *SNURF/IPW* domain on 15q11-q13. PEGs are shown as blue rectangles, and MEGs are shown as pink rectangles. miRNAs and snoRNAs (small nucleolar RNAs) are depicted as stem loops and ovals, respectively. Arrows indicate the direction of transcription. The DMRs ICR1, IG-DMR, *MEG3*-DMR, and IC, which control monoallelic expression over the domains, are indicated by closed and open lollipops (methylated and unmethylated, respectively). SGA, small for gestational age.

Genomic imprinting is a physiological process defined as the monoallelic expression of a gene according to its parental origin, under the control of a differentially methylated region (DMR), known as the imprinting control region (ICR) (*1*). More than 150 human genes have been shown to be imprinted. Imprinting disorders (IDs), caused by disturbances of imprinted genes, are a group of congenital diseases affecting growth, development, and metabolism in humans, leading to diseases with overlapping features, regardless of the genomic region affected (*2*). Some of this overlap may be explained by the co-regulation of imprinted genes, which belong to an imprinted gene network (IGN) involved in the control of cellular proliferation and differentiation (*3*). Recent studies in mammals have shown how the disturbance of one imprinted gene can affect other maternally expressed genes (MEGs) or paternally expressed genes (PEGs) (*4*–*6*). The overlap between Silver-Russell syndrome (SRS) (*7*) and Temple syndrome (TS14) (*8*) is a particularly demonstrative example of clinical overlap between IDs. Both these syndromes include fetal and postnatal growth retardation, early feeding difficulties, early puberty, and an increase in the risk of metabolic disorders (Fig. 1A) (*8*, *9*). Moreover, TS14 patients also have a number of clinical features in common with another ID, Prader-Willi syndrome (PWS), a differential diagnosis for TS14 (Fig. 1A) (*8*). Most SRS patients carry molecular changes in the 11p15.5 region (Fig. 1B), the most prevalent (~50%) of which is hypomethylation of the DMR *H19/IGF2*:IG-DMR (hereinafter referred to as ICR1), decreasing paternal *IGF2* (a potent fetal growth factor) expression and increasing the maternal expression of *H19* (*10*), encoding a lncRNA. TS14 patients present molecular abnormalities at the paternally methylated imprinted locus on chromosome 14q32.2 (Fig. 1C). The most frequent of these abnormalities is maternal uniparental disomy or upd(14)mat. Paternal deletions of the imprinted *DLK1/MEG3* domain and *DLK1/MEG3*:IG-DMR (hereinafter referred to as IG-DMR) hypomethylation are less frequent (*8*). Last, PWS patients present disturbances of imprinted genes at the *SNRPN* locus and its ICR (IC-*SNRPN*) in the 15q11-q13 region (Fig. 1D), resulting in a loss of the expression of *SNRPN/SNURF* and *IPW*, two PEGs mapping to the minimal deletion interval critical for PWS (*6*). A recent clinical study published in 2017 on a large cohort of 32 TS14 patients with 14q32.2 genetic- and epigenetic-related defects revealed both PWS- and SRS-like phenotypes in 50% of patients (*11*). Moreover, we have recently shown that chromosome 14q32.2 imprinting defects are an alternative molecular diagnosis of SRS (*12*). Evidence is accumulating that these methylation defects in patients with SRS and TS14 are not isolated events, with some patients having multilocus imprinting disturbances (MLIDs) affecting additional imprinted regions (*12*, *13*).

Human chromosome 14q32.2 encompasses an imprinted region containing three PEGs (*DLK1*, *RTL1*, and *DIO3*) and a number of MEGs. All the MEGs encode noncoding RNAs (ncRNAs) (*MEG3*/*GTL2*, *MEG8*, *MEG9*, and *RTL1AS*) and several large clusters of microRNAs (miRNAs) and small nucleolar RNAs (snoRNAs) (*14*). The monoallelic parent-specific expression of these genes is controlled by the germline–derived primary intergenic IG-DMR and the postfertilization-derived secondary *MEG3*-DMR, both of which are methylated on the paternal allele and unmethylated on the maternal allele (*15*). PEGs from the 14q32.2 region play a crucial role in cell differentiation and tissue development, whereas the function of the MEGs remains unclear (*16*). It has been shown that the hypermethylation of the IG-DMR results in the reactivation of the normally silenced maternal allele of PEGs and a loss of expression of MEGs (*17*). The effect of ICR1 hypomethylation on the expression of *IGF2/H19* domain genes has been determined for SRS patients (*10*), but the effect of IG-DMR hypomethylation has yet to be determined in TS14 patients.

**Fig. 2:**
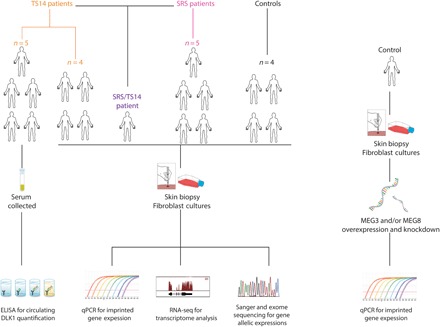
Schematic presentation of the patients, biological materials, and strategies used in the study. ELISA, enzyme-linked immunosorbent assay; qPCR, quantitative polymerase chain reaction; RNA-seq, RNA sequencing.

We studied gene expression following the hypomethylation of IG-DMR to characterize the effect of this epigenetic alteration on *DLK1/MEG3* domain gene expression. We performed expression profiling for imprinted and nonimprinted genes in human fibroblasts from TS14 (IG-DMR hypomethylation) and SRS (ICR1 hypomethylation) patients (Fig. 2) to identify possible gene expression signatures common to these two IDs, which present a major clinical overlap.

## RESULTS

### TS14 patients’ molecular diagnostics and collected biological materials

We collected serum from seven TS14 patients with IG-DMR and *MEG3*-DMR hypomethylation (*n* = 5) or 14q32.2 paternal deletion (*n* = 2). We also established fibroblast cell cultures for four TS14 patients with IG-DMR hypomethylation, one SRS/TS14 patient with both 11p15.5 ICR1 and IG-DMR hypomethylation, five SRS patients with ICR1 hypomethylation, and five controls (cells were provided by Coriell Cell Repositories). Clinical data and methylation levels for all patients and controls are listed in tables S1 and S2, respectively.

### DLK1 is absent from the serum of TS14 patients but present in that of age-matched controls

**Fig. 3:**
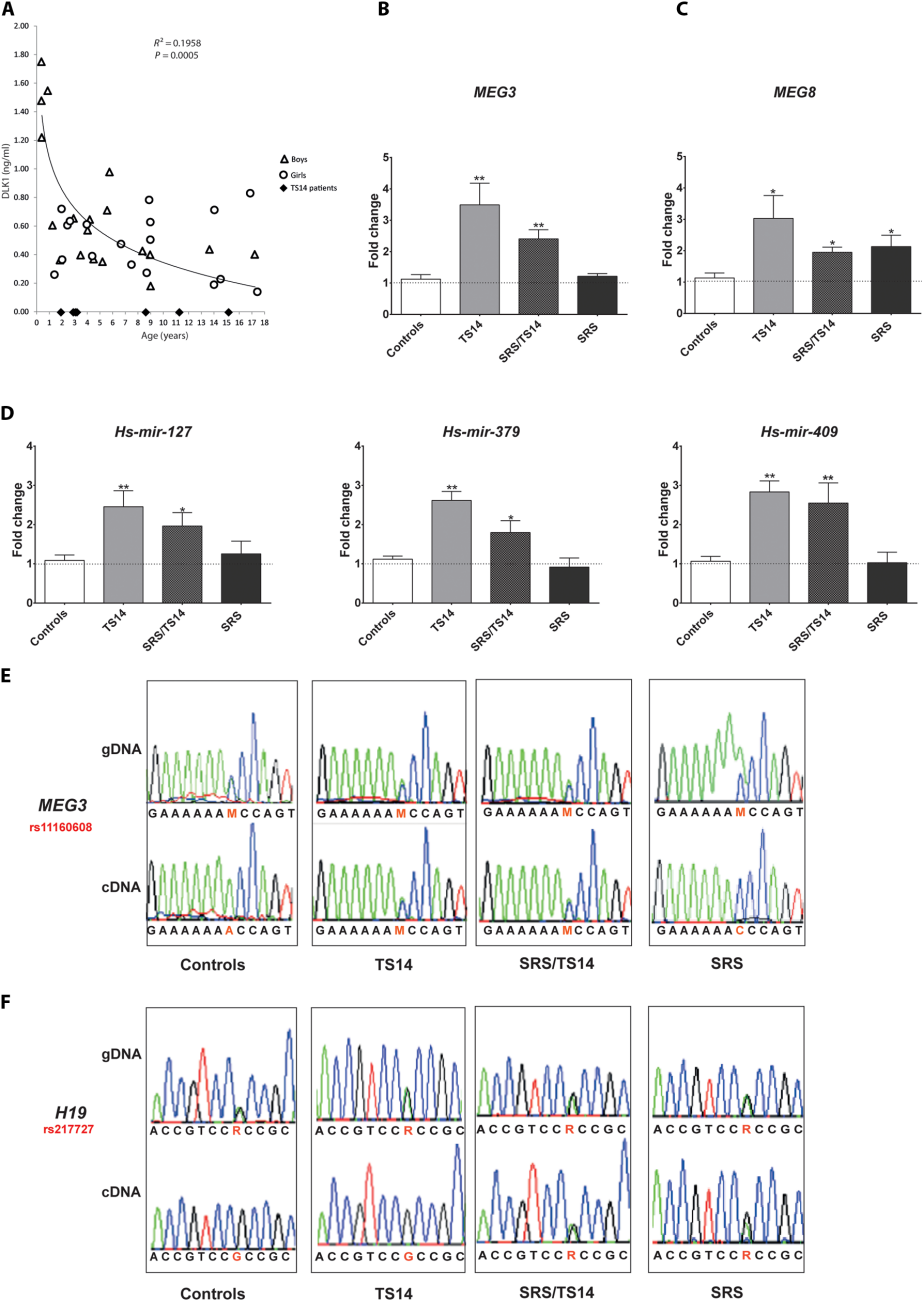
Expression profiling of 14q32.2 genes from the serum and fibroblasts of TS14 patients. (**A**) DLK1 is absent from the serum of TS14 patients but present in that of age-matched controls. Boys and girls are indicated by open triangles and circles, respectively. TS14 patients are represented by black diamonds. (**B** to **D**) *MEG3*, *MEG8*, and miRNAs are up-regulated in TS14 patient fibroblasts. Relative levels of expression for *MEG3*, *MEG8*, and three miRNAs of the *DLK1/MEG3* domain in skin-derived fibroblast cultures from TS14, SRS/TS14, and SRS patients, compared with control fibroblasts. (**E** and **F**) *MEG3* and *H19* are biallelically expressed upon the hypomethylation of 14q32.2 and 11p15.5, respectively. Electropherogram showing the informative SNPs rs11160608 and rs217727, within the *MEG3* (E) and *H19* (F) coding sequences, respectively, on genomic DNA (gDNA) and cDNA. The data shown are mean values ± SEM for five different passages for the SRS/TS14 patient skin-derived fibroblast cultures, four TS14 patients and five SRS patients, with comparison to five donors as a control. *P ≤ 0.05 and **P ≤ 0.01 versus controls, in Mann-Whitney tests.

DLK1 is a single-pass transmembrane protein that can be cleaved by extracellular proteases to release a circulating form (*18*). We assessed the effect of *DLK1/MEG3* domain hypomethylation on *DLK1* expression by first measuring the circulating levels of DLK1 in the serum of healthy children (*n* = 38, 19 boys and 19 girls) between the ages of 0 and 17 years. We found that serum DLK1 levels decreased considerably after birth, but those patients with paternal deletions or hypomethylation of the *DLK1/MEG3* domain had barely detectable levels of DLK1, regardless of their sex, age, or the molecular defect at 14q32.2 (Fig. 3A).

### MEG3, MEG8, and miRNAs are up-regulated because of their expression from the normally silenced paternal allele

We then investigated the levels of transcription of 14q32.2 MEGs in cultured fibroblasts from TS14 and SRS patients (PEGs were not expressed in fibroblasts). We found that the levels of the lncRNA *MEG3* and the ncRNA *MEG8* in all TS14 and SRS/TS14 fibroblasts were at least twice those in fibroblasts from controls (Fig. 3, B and C). SRS fibroblasts had normal *MEG3* levels, but *MEG8* levels were twice those in control fibroblasts (Fig. 3, B and C). In addition to *MEG3* and *MEG8*, we quantified nine miRNAs (encoded by genes distributed throughout the 14q32.2 region), all of which were found to be up-regulated in fibroblasts from TS14 and SRS/TS14 patients relative to SRS patients and controls (Fig. 3D shows data for three of the nine miRNAs). We assessed the stability and passage independence of the overexpression of these genes by quantifying the mRNAs at five different passages for each patient. We found that, in all patients, all the ncRNAs and miRNAs studied were stably overexpressed in all cultures (fig. S1, A and B). Furthermore, single-nucleotide polymorphism (SNP) genotyping (Fig. 3E) provided the first evidence of biallelic expression of *MEG3* in fibroblasts from patients with TS14, due to reactivation of the normally silenced paternal allele. No SNP assessment was possible for *MEG8* or the miRNAs.

### MLIDs at 14q32.2 and 11p15.5 are associated with multi-imprinting relaxation

Fibroblasts from a patient with SRS/TS14 displaying hypomethylation at both 14q32.2 and 11p15.5 loci displayed biallelic expression of *MEG3* from the *DLK1/MEG3* domain. We investigated whether MLID induced multi-imprinting relaxation and biallelic expression at the secondary loci affected by sequencing *H19* genomic DNA (gDNA) and complementary DNA (cDNA) from SRS/TS14 fibroblasts. We found that *H19* was monoallelically expressed in TS14 fibroblasts, whereas it was biallelically expressed in SRS and SRS/TS14 fibroblasts (Fig. 3F).

### 11p15.5 and 15q11-q13 PEGs are down-regulated by 14q32.2 hypomethylation

**Fig. 4:**
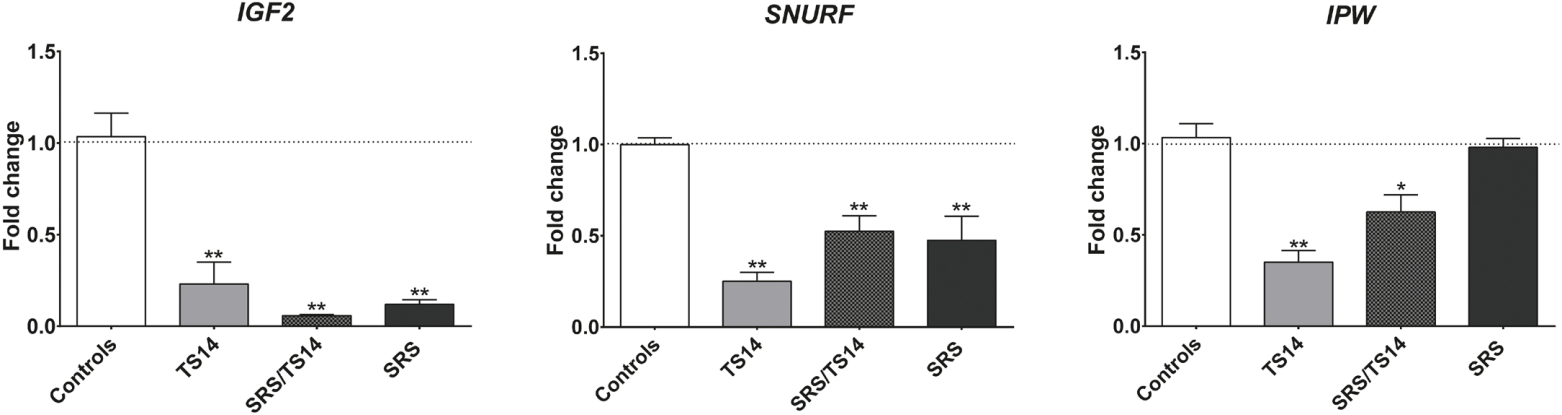
11p15.5 and 15q11-q13 PEGs are down-regulated upon 14q32.2 hypomethylation. Relative levels of expression for *IGF2*, *SNURF*, and *IPW* in cultures of skin-derived fibroblasts from TS14, SRS/TS14, and SRS patients compared with control fibroblasts. The data shown are mean values ± SEM for five different passages for the skin-derived fibroblast cultures for the SRS/TS14 patient, four TS14 patients, and five SRS patients, with comparison to five different donors as a control. *P ≤ 0.05 and **P ≤ 0.01 versus controls, in Mann-Whitney tests.

We then studied the expression of a large number of imprinted genes from loci involved in growth control in TS14, SRS, and control fibroblasts. For most of the PEGs, expression was barely detectable (*PEG3*, *PEG10*, and *GNAS-XLa*), whereas MEGs were highly variable (*H19*) or comparable to controls (*UBE3A* and *GRB10*) in fibroblast cultures (fig. S1C). We found that the 11p15.5 (*IGF2*) and 15q11-q13 (*SNURF* and *IPW*) PEGs were strongly and stably expressed in cultures of fibroblasts from controls and patients. All TS14 fibroblasts displayed low levels of *IGF2* expression, as did SRS and SRS/TS14 fibroblasts. Furthermore, both TS14 and SRS fibroblasts displayed low levels of *SNURF* expression, whereas only TS14 fibroblasts had low levels of *IPW* expression (Fig. 4). Similar to the ncRNAs and miRNAs, *IGF2*, *SNURF*, and *IPW* were all stably down-regulated in all fibroblast cultures, at various passages, for all patients (fig. S1D).

### MEG3 and MEG8 overexpression and knockdown in control fibroblasts are associated with deregulation of the expression of 11p15.5 and 15q11-q13 PEGs

To study whether the clinical overlap could be explained by the cross-regulation between the three imprinted regions, we investigated the possible role of 14q32.2 ncRNAs *MEG3* and *MEG8* in the changes in *IGF2*, *SNURF*, and *IPW* expression. For this purpose, we carried out overexpression and knockdown of MEG3 and/or MEG8 in a control primary fibroblast line.

Expression constructs were available only for *MEG3*. For *MEG8*, we inserted the full-length *MEG8* cDNA obtained from fibroblasts into an expression vector. *MEG8* cDNA amplification revealed the presence of two different transcripts: a 497-base pair (bp) transcript corresponding to NR_024149.2 and a previously unreported 639-bp transcript (GenBank KX237564; fig. S2). This new variant has an additional exon, located between exons 1 and 2 of the NR_024149.2 transcript, flanked by donor and acceptor sites for splicing.

**Fig. 5:**
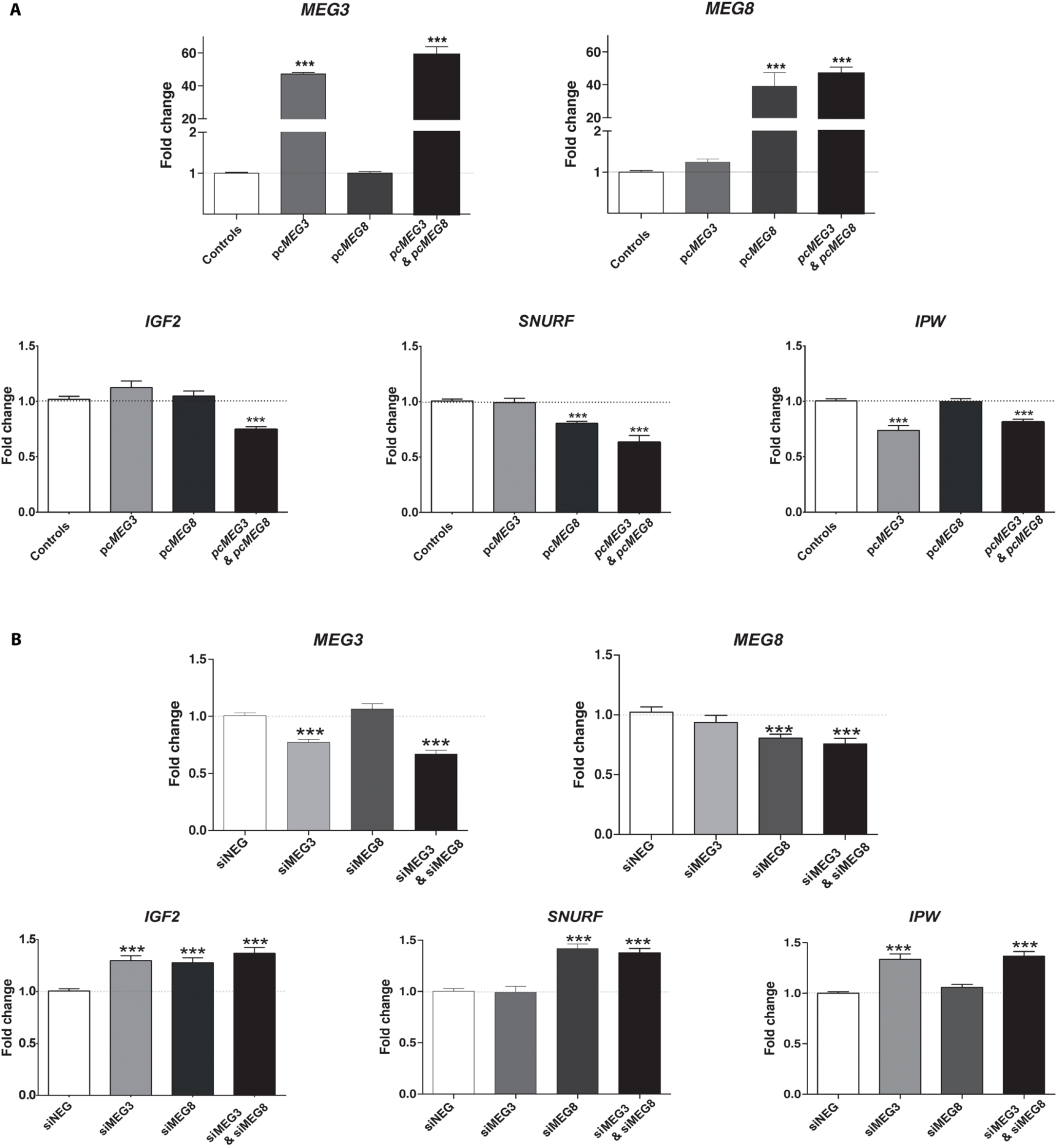
11p15.5 and 15q11-q13 PEGs are deregulated upon *MEG3* and/or *MEG8* overexpression or knockdown in experimental models. (**A**) Relative levels of expression for *IGF2*, *SNURF*, and *IPW* in a control primary fibroblast line transfected with an empty, *MEG3*, and/or *MEG8* expression vector. (**B**) Relative levels of expression for *IGF2*, *SNURF*, and *IPW* for a control primary fibroblast line transfected with a negative control, *MEG3*, and/or *MEG8* small interfering RNA (siRNA). The data shown are the mean values ± SEM for four independent transfection assays (with at least four wells transfected per experiment). ***P ≤ 0.001 versus controls (transfected with an empty vector), in unpaired *t* tests.

We found that the concomitant overexpression of *MEG3* and *MEG8* in control fibroblast cultures was associated with a moderate but significant down-regulation of *IGF2* expression (Fig. 5A). As for the 15q11-q13 PEGs, we found that overexpressing *MEG8* (regardless of the variant used for transfection) in control fibroblasts was associated with a down-regulation of *SNURF* expression, whereas *MEG3* overexpression was associated with *IPW* down-regulation (Fig. 5A). To investigate whether the down-regulation of *MEG3* and *MEG8* could also lead to deregulation of theses PEGs, we knocked down both ncRNA expression with *siRNA* in the same cell line. We found that *IGF2* expression increased following *MEG3* and/or *MEG8* knockdown, whereas *IPW* and *SNURF* were up-regulated by the knockdown of *MEG3* and *MEG8*, respectively (Fig. 5B). These changes in the expression of *IGF2*, *SNURF*, and *IPW* were observed in all transfected cells, despite the low transfection efficiency due to the use of primary fibroblast cultures as the host cells for transfection assays.

### Principal components analysis of overall gene expression in TS14 and SRS fibroblasts

**Fig. 6:**
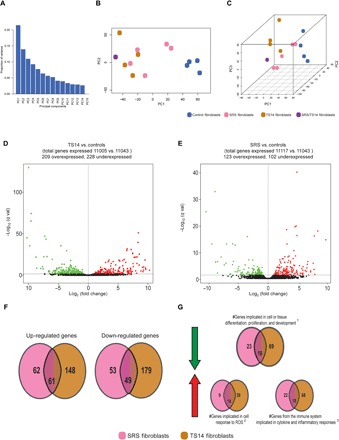
PCA and DEGs from TS14, SRS, and control fibroblasts. (**A**) Proportion of the variance accounted for by each principal component (PC). (**B**) Two-dimensional and (**C**) three-dimensional projection plots of the first three PCs: PC1, PC2, and PC3. (**D** and **E**) Volcano plots of the expression of all genes (with FPKM of ≥1) from TS14 and SRS, respectively, relative to controls. Green and red dots represent gene displaying statistically significant underexpression and overexpression, respectively [log_2_(±2) fold change, with a *q* value threshold of 0.05]. (**F**) The number of genes differentially regulated in both TS14 and SRS fibroblasts or in one of these types of fibroblasts only. (**G**) Schematic representation of gene ontology pathways from the GSEA displaying significant up- and down-regulation in patients relative to controls fibroblasts after correction for multiple testing (*q* < 0.05). GSEA reveals a dysregulated pathway signature common to TS14 and SRS.

We investigated the dynamic changes in global gene expression levels and searched for possible gene expression signatures common to these two IDs by performing RNA sequencing (RNA-seq)–based expression profiling on TS14, SRS, and control fibroblasts [Gene Expression Omnibus (GEO) accession number GSE109408]. We first analyzed and compared the overall gene expression patterns of TS14 and SRS fibroblasts. For this purpose, we used principal components analysis (PCA), a statistical technique that summarizes large datasets, reducing the number of dimensions while illustrating relationships between samples based on the covariance of the data considered (*19*). PC1, PC2, and PC3 accounted for ~50% of the total variance of the original data (Fig. 6A). When all samples (TS14, SRS, and controls) were plotted in a two-dimensional space defined by PC1 and PC2 (accounting for ~35% of all variation), we were able to distinguish two distinct groups, the first clustering all the TS14 and SRS patients and the second encompassing all the controls (Fig. 6B). However, when the samples were plotted in a three-dimensional space defined by PC1, PC2, and PC3, we were able to separate the TS14 + SRS group into two separate groups, one corresponding to TS14 and the other to SRS (Fig. 6C). This analysis provides the first evidence for overlapping but distinguishable gene expression profiles in TS14 and SRS patients.

### RNA-seq reveals that imprinting defects lead to the genome-wide deregulation of gene expression

We evaluated differential gene expression patterns in TS14 and SRS fibroblasts by grouping our samples based on their imprinting defects: 14q32.2 hypomethylation (four TS14 patients), 11p15.5 hypomethylation (five SRS patients), and controls (four donors). For this analysis, we selected genes with at least a twofold difference in expression between patients and controls and a *q* value below 0.05. We increased stringency by considering only genes with a mean expression of ≥1 FPKM (fragments per kilobase million) in at least one group. Using these criteria, we identified a total of 11,005, 11,117, and 11,043 expressed genes in control, SRS, and TS14 fibroblasts, respectively. Out of them, 552 genes were differentially expressed genes (DEGs), with expression levels differing between control fibroblasts and fibroblasts obtained from patients with TS14 or SRS (Fig. 6, D and E). Similar numbers of genes were up-regulated (271 genes) and down-regulated (281 genes). We found that 61 of the up-regulated genes were up-regulated in both TS14 and SRS fibroblasts, whereas 62 were up-regulated only in SRS fibroblasts, and 148 were up-regulated only in TS14 fibroblasts. By contrast, 49 of the down-regulated genes were down-regulated in both TS14 and SRS fibroblasts, whereas 53 were down-regulated only in SRS fibroblasts, and 179 were down-regulated only in TS14 fibroblasts (Fig. 6F). Together with the quantitative polymerase chain reaction (qPCR) findings in patients and transfected normal fibroblasts, these results show that imprinting defects induce the deregulation of both imprinted and nonimprinted genes. Moreover, 50% of the DEGs in SRS fibroblasts was also deregulated in TS14 fibroblasts (Fig. 6F). The list of all the DEGs in SRS and TS14 fibroblasts is available in data file S1.

### A gene signature common to TS14 and SRS identified by gene set analysis

We then determined whether networks of genes with similar functional annotations were dysregulated in TS14 and SRS patients relative to controls. We analyzed the genes displaying differential expression between these groups using gene ontology pathways from gene set enrichment analysis (GSEA) to annotate genes on the basis of the functional biological processes in which their products are involved. GSEA with a false discovery rate (*q* value) threshold of *q* < 0.05, to correct for multiple testing, identified one major pathway as down-regulated and two as up-regulated in both TS14 and SRS (Fig. 6G: ^1^Go Cell Development, Go Regulation Of Cell Differentiation, Go Positive Regulation Of Developmental Process, Go Tissue Development. ^2^Go Cellular Response To Organic Substance, Go Response To Oxygen Containing Compound. ^3^Go Immune System Process, Go Response To External Stimulus, Go Cytokine Mediated Signaling Pathway, Go Inflammatory Response.). We found that the level of gene expression was lower (87 genes for TS14 and 41 genes for SRS) in both TS14 and SRS fibroblasts for genes encoding products known to enhance cell or tissue proliferation, growth, and development, whereas genes from the immune system involved in inflammatory, cytokine, and reactive oxygen species reactions were overexpressed. In all cases, TS14 fibroblasts had three to four times more DEGs from these pathways than SRS fibroblasts. Once again, 50% of the DEGs in SRS was also found among the DEGs in TS14 dysregulated pathways.

### Allele-specific expression of imprinted genes from TS14 and SRS/TS14 fibroblasts

We previously performed whole-exome sequencing (WES) on DNA extracted from the fibroblasts of two TS14 patients (TS14-1 and TS14-2) (*12*) and the SRS/TS14 patient. We then used a method combining RNA-seq and WES of these patients to accurately assess allele-specific expression (ASE; fig. S3). On average, 3825 genes were found to be expressed biallelically versus 103 expressed monoallelically (3941, 3638, and 3895 versus 105, 122, and 82 from TS14-1, TS14-2, and SRS/TS14 patients, respectively). We first focused on imprinted human genes from the list of genes available from the geneimprint database (www.geneimprint.com). We compared their ASE to the bulk/single-cell fibroblast ASE recently reported for five healthy individuals (table S3) (*20*). Consistent with their known imprinted status, we found that four imprinted genes had monoallelic expression in both patient and control fibroblasts (*OSBPL5*, *NDN*, *ZDBF2*, and *PEG10*). The *H19* gene was found to be monoallelically expressed in patient TS14-1, whereas the *PRIM2* gene, predicted to be imprinted on the basis of previous studies, also displayed monoallelic expression in both TS14 patients. However, we identified 16 other imprinted genes with biallelic expression in our patients’ fibroblasts (table S3). Thirteen of these genes were also biallelically expressed in bulk fibroblast cultures from controls, suggesting that these genes may not be imprinted in fibroblasts. For the remaining three genes, *CPA4* was biallelically expressed in all three patients, whereas *ZNF597* and *H19* (consistent with our Sanger sequencing results for cDNA) were biallelically expressed only in the SRS/TS14 patient.

Fibroblasts from the SRS/TS14 patient displaying hypomethylation at both 14q32.2 and 11p15.5 loci displayed biallelic expression of *MEG3* and *H19* from the *DLK1/MEG3* and *IGF2/H19* domains, respectively. We investigated whether the biallelic expression of *CPA4* and *ZNF597* also resulted from MLID by assessing the methylation levels of the two imprinted regions regulating these genes (the *PEG1/MEST* and *ZNF597*-DMRs, respectively). The *PEG1/MEST* locus was hypomethylated in all three patients, whereas *ZNF597* was hypomethylated only in the SRS/TS14 patient, consistent with the biallelic expression profiles of these patients (table S4). These results confirm our previous finding for MLID and multi-imprinting relaxation together with biallelic expression at the affected secondary loci. However, as both *CPA4* and *ZNF597* are expressed from the methylated maternal allele, their biallelic expression upon hypomethylation of their respective domains is discordant with their parent-of-origin expression. Such discordances have been reported for other imprinted genes but never before for these two genes (*20*). Last, we looked at the remaining genes of unknown imprinting status displaying monoallelic expression (137 genes; table S4) in our patients. We identified seven putative imprinted genes clustering at four different loci across the genome (fig. S4). These variants were not validated by another targeted sequencing technique.

## DISCUSSION

IDs often affect growth in humans, leading to diseases with overlapping features, regardless of the genomic region affected (*2*). We characterized the effect of a loss of methylation at the imprinted *DLK1/MEG3* locus on gene expression at the 14q32.2 locus in TS14 patients, and we investigated whether the overlap in clinical phenotype between TS14 and SRS and PWS might be due to similar patterns of gene deregulation between these syndromes.

*DLK1* is an important PEG in the 14q32.2 domain. This gene encodes a transmembrane protein that generates both membrane-bound and serum-soluble isoforms through alternative splicing (*16*). We found that TS14 patients with paternal deletions or hypomethylation of the *DLK1/MEG3* domain had no circulating DLK1, whereas serum DLK1 concentration is negatively correlated with age after birth in healthy children [consistent with the rapid decrease in serum Dlk1 concentration observed in newborn mice (*21*)]. Thus, unlike circulating IGF2, which is produced biallelically in the liver after birth, under the control of a specific nonimprinted promoter (*22*), circulating *DLK1* probably continues to be generated by monoallelic expression from the imprinted gene under normal conditions after birth. An analysis of the MEGs from the 14q32.2 domain showed that *MEG3*, *MEG8*, and miRNAs were up-regulated in TS14 fibroblasts. We demonstrated that *MEG3* overexpression resulted from the reactivation of the normally silenced paternal allele. We hypothesized that the overexpression of *MEG8* and miRNA was also due to expression from both parental alleles in TS14 patients. This hypothesis is consistent with the finding of previous studies that *DLK1/MEG3* hypermethylation leads to a reactivation of the expression of *RTL1* and *DLK1* from the maternal allele (*17*). These findings highlight the parent-of-origin–specific effects of DNA methylation at ICRs on gene expression in the *DLK1/MEG3* domain.

We investigated the possible overlap in gene expression patterns between TS14 and SRS and between TS14 and PWS. We found that imprinted genes within the primary affected domains for SRS (*IGF2* at 11p15.5) and PWS (*SNURF* and *IPW* at 15q11-q13) were down-regulated in all TS14 fibroblasts and in SRS/TS14 fibroblasts, despite the normal levels of methylation at their imprinted control regions, that are generally altered in SRS and PWS patients (*6*, *23*). We hypothesized that this deregulation of the IGN might affect gene expression throughout the genome. An unsupervised PCA on the RNA-seq data from control and TS14 and SRS patient fibroblast cultures resulted in the clustering of samples into two groups (controls on the one hand and SRS and TS14 patients on the other hand) when the data were projected onto a two-dimensional space. However, the SRS and TS14 patients could be distinguished when the data were projected onto a three-dimensional space. Gene ontology annotations showed that the genes displaying expression alterations belonged to common pathways. Genes encoding products that promote cell and tissue growth were found to be down-regulated in fibroblasts from both TS14 and SRS patients. These results confirm our hypothesis of an overlapping but distinguishable, genome-wide deregulation of the pattern of expression in TS14 and SRS patients, affecting not only other imprinted genes from the IGN but also a number of nonimprinted genes. We showed that hypomethylation at the *DLK1/MEG3* domain leads to the down-regulation of other imprinted PEGs, such as *IGF2*, *SNURF*, and *IPW*, mostly genes known to enhance growth (*24*), and of 87 other nonimprinted genes implicated in promoting growth. Moreover, TS14 fibroblasts had about twice as many deregulated (1.6× up- and 2.2× down-regulated) genes as SRS fibroblasts, with about 50% of the deregulated genes (61 up- and 49 down-regulated) common to both syndromes, regardless of the imprinting defect. This finding may partly explain the phenotypic overlap and also some of the differences between SRS and TS14, such as the much early onset of puberty and greater early weight gain in TS14 than in SRS patients.

Last, we hypothesized that these changes in expression might reflect, directly or indirectly, the involvement of 14q32.2 ncRNAs *MEG3* and *MEG8* in this process. ncRNAs have been shown to regulate gene expression both in cis and in trans (*25*, *26*), through association with chromatin modifiers (*27*). This raises the possibility of 14q32.2 ncRNAs regulating the expression of these genes from the 11p15.5 and 15q11-q13 regions. We found that overexpressing or knocking down the expression of *MEG3* and *MEG8* in normal fibroblast cultures was associated with deregulation of PEGs from the 11p15.5 and 15q11-q13 regions. We found in our experimental models of normal fibroblasts that (i) overexpressing *MEG3* was associated with decreased level of *IPW* transcripts, (ii) overexpressing *MEG8* was associated with lower levels of *SNURF*, and (iii) concomitant overexpression of *MEG3* and *MEG8* was associated with lower levels of *IGF2* transcripts. These results are in accordance with observations in TS14 and SRS fibroblasts, where (i) *IPW* levels were lower only in TS14 fibroblasts where *MEG3* was overexpressed; (ii) *SNURF* levels were lower in TS14 and SRS patients, both having increased levels of *MEG8*; and (iii) *IGF2* levels were lower in TS14 patients overexpressing *MEG3* and *MEG8* upon *DLK1/MEG3* hypomethylation. These results suggest that *MEG3* and *MEG8* can regulate, in trans, the expression of other imprinted genes. We hypothesize a synergic role of MEG3 and MEG8 to control directly or indirectly the expression of *IGF2*. Overexpressing one of the two is not enough to increase *IGF2* expression, since the other component is missing, while knocking down only one is enough to disrupt the complex and lead to decreased *IGF2* expression. Similar patterns of regulation within the *IGN* have been described before (*3*, *5*), involving *IPW* and *MEG3* in particular (*6*), for which *IPW* was found to repress *MEG3* expression. Together with our results, this strongly suggests that a system of reciprocal control operates between ncRNAs at imprinted loci and that this system might contribute to the clinical overlap between IDs. However, additional functional studies of the role of *MEG3* and *MEG8* are required to decipher the mechanisms by which these ncRNAs could regulate gene expression, particularly for imprinted genes. These mechanisms are probably unrelated to gene imprinting status because all TS14 patients have normal methylation levels at the ICRs corresponding to the deregulated imprinted genes (ICR1 for *IGF2* and *SNRPN*-IC for *SNURF* and *IPW*). Single-cell RNA-seq has been shown to be more powerful than bulk RNA-seq for studies of the expression of imprinted genes (*20*) and therefore would allow the generation of a more extensive map of the deregulated IGN upon hypomethylation of the *DLK1/MEG3* and/or *IGF2/H19* domains.

In conclusion, we show that 14q32.2 hypomethylation affects the expression not only of genes within this locus but also of other imprinted and nonimprinted genes, some of which are involved in controlling tissue growth. We also observed changes in gene expression common to TS14 and SRS patients, in particular, a diminished *IGF2* expression, and to TS14 and PWS patients, with diminished *SNURF* and *IPW* expressions (fig. S5). This transcriptomic overlap may account for the clinical overlap between the two syndromes.

## MATERIALS AND METHODS

### Study approval

Patients were followed up at Armand Trousseau Children’s Hospital or were referred by other clinical centers for molecular analysis for suspected TS14 or SRS. A geneticist and/or a pediatric endocrinologist examined each patient, and a detailed clinical form was completed. All patients or their parents and the controls included in this study gave written informed consent for participation, in accordance with French national ethics rules for patients recruited in France (Assistance Publique–Hôpitaux de Paris authorization no. 682) and the rules of Institutional Review Board I00000204 of the Mount Sinai School of Medicine for patients recruited in the United States.

### Quantification of serum DLK1 levels

Serum DLK1 levels from healthy children and TS14 patients were quantified in 96-well plates, with the Quantikine Immunoassay Control for Human Pref-1/DLK1/FA1 (R&D Systems, France), based on sandwich ELISA (enzyme-linked immunosorbent assay), according to the manufacturer’s instructions.

### Skin-derived fibroblast cultures

Skin-derived fibroblasts from patients and controls were cultured to confluence in RPMI 1640 (Gibco, Cergy Pontoise, France) supplemented with 10% fetal calf serum and ampicillin (50 U/ml)/streptomycin (50 μg/ml) at 37°C (number of passages, <10). Cells were then treated with trypsin and centrifuged. The cell pellet obtained was washed twice with 1× phosphate-buffered saline and used for DNA and micro/large-scale RNA extractions.

### Nucleic acid extraction and quantification

DNA was extracted from blood leukocytes and from skin-derived fibroblast cultures, by an in-house protocol, after cell lysis by a salting-out procedure, as previously described (*28*, *29*). Total RNA and miRNA were extracted from cultured fibroblasts with the NucleoSpin miRNA Kit for the isolation of small and large RNAs (MACHEREY-NAGEL, France). Both DNA and RNA were quantified with a NanoDrop ND-1000 spectrophotometer (Invitrogen, France).

### Reverse transcription and real-time PCR quantification of micro/mRNAs

We synthesized cDNA from micro/mRNAs isolated from fibroblasts and used it for quantitative PCR with the miScript PCR System (Qiagen, France). Expression in the controls was arbitrarily set to 1, and fold changes (FCs) between two groups were calculated as FC = 2^−ΔΔ*C*t^. Details from the reverse transcription and real-time quantification analysis are provided in Supplementary Methods.

### Bisulfite treatment of DNA and methylation analysis

gDNA (1 μg) was treated with sodium bisulfite, with the EZ DNA Methylation Kit (Zymo Research, Orange, CA), according to the manufacturer’s instructions. The methylation status of the studied loci was assessed by allele-specific methylated multiplex real-time quantitative PCR (ASMM RTQ-PCR), as previously described (*30*–*36*). Details for the treatment and technique are provided in Supplementary Methods.

### gDNA and cDNA sequencing

Two SNPs from the *MEG3* and *H19* gDNA and cDNA were sequenced by standard Sanger sequencing methods, with the ABI PRISM BigDye Terminator v3.0 Cycle Sequencing Kit and the ABI 3100 Genetic Analyzer. The sequencing products were then analyzed with SeqScape v2.6 (Life Technologies, Courtaboeuf, France).

### Cell cultures and transfection assays

GM05757 (control human skin–derived fibroblast) cells were cultured in 24-well plates for overexpression and small interfering RNA (siRNA) transfections. Details of culture and transfection conditions and constructs used are detailed in Supplementary Methods.

### WES, RNA-seq, and bioinformatics analysis

Library preparations, sequencing, and data analysis were carried out by IntegraGen SA (Evry, France). The sequencing methods and bioinformatics analysis are described in detail in Supplementary Methods.

### RT-qPCR statistical analysis

For RT-qPCR, we compared data for pairs of groups in Mann-Whitney tests (expression in fibroblasts from patients versus that in control fibroblasts, with *n* = 5 each) and unpaired *t* tests (overexpression and siRNA assays with *n* > 20 per condition). We considered *P* values below 0.05 to indicate statistical significance. The analyses were performed with GraphPad Prism version 6.00 (GraphPad Software, La Jolla, CA, USA).

## Supplementary Material

http://advances.sciencemag.org/cgi/content/full/5/2/eaau9425/DC1

## SUPPLEMENTARY MATERIALS

Supplementary material for this article is available at http://advances.sciencemag.org/cgi/content/full/5/2/eaau9425/DC1 Supplementary Methods Fig. S1. Expression of 14q32.2 MEGs and 11p15.5 and 15q11-q13 PEGs in five different passages of cultured fibroblasts from TS14 patients. Fig. S2. Schematic representation of the subcloned transcripts of MEG8 (*MEG8a* and *MEG8b*). Fig. S3. Distribution of minor allele frequency according to combined exome and RNA-seq data for fibroblasts from the TS14-1, TS14-2, and SRS/TS14 patients. Fig. S4. Schematic representation of the four putative imprinted loci identified on the basis of allele-specific gene expression and DMRs. Fig. S5. Schematic representation of the molecular findings and the hypothesized mechanism from this study. Table S1. Methylation levels for all patients and controls, as determined by ASMM RT-qPCR. Table S2. Clinical features for TS14 patients with imprinting defects at the *DLK1/MEG3* domain described here. Table S3. The allelic status of imprinted genes, as determined by RNA and exome sequencing for TS14 and control fibroblasts. Table S4. List of all genes with monoallelic expression, as determined by RNA and exome sequencing in TS14 patients. Table S5. List and sequences of the primers used in this study. Data file S1. List of all gene FPKMs for each patient and control. Data file S2. Supervised study of DEGs for each group of patients compared to controls. References (*37*–*40*)
