# Supplementary material for: Transcriptional profiling at the DLK1/MEG3 domain explains clinical overlap between imprinting disorders
Source: Sci Adv. 2019 Feb 20;5(2):eaau9425. doi: 10.1126/sciadv.aau9425 (PMC6382400; doi:10.1126/sciadv.aau9425)
Supplement: http://advances.sciencemag.org/cgi/content/full/5/2/eaau9425/DC1 [file supp_5_2_eaau9425__index.html]

Science Advances | Science Advances

## Supplementary Materials

**The PDF file includes:**

- Supplementary Methods
- Fig. S1. Expression of 14q32.2 MEGs and 11p15.5 and 15q11-q13 PEGs in five different passages of cultured fibroblasts from TS14 patients.
- Fig. S2. Schematic representation of the subcloned transcripts of MEG8 (*MEG8a* and *MEG8b*).
- Fig. S3. Distribution of minor allele frequency according to combined exome and RNA-seq data for fibroblasts from the TS14-1, TS14-2, and SRS/TS14 patients.
- Fig. S4. Schematic representation of the four putative imprinted loci identified on the basis of allele-specific gene expression and DMRs.
- Fig. S5. Schematic representation of the molecular findings and the hypothesized mechanism from this study.
- Table S1. Methylation levels for all patients and controls, as determined by ASMM RT-qPCR.
- Table S2. Clinical features for TS14 patients with imprinting defects at the *DLK1/MEG3* domain described here.
- Table S3. The allelic status of imprinted genes, as determined by RNA and exome sequencing for TS14 and control fibroblasts.
- Table S4. List of all genes with monoallelic expression, as determined by RNA and exome sequencing in TS14 patients.
- Table S5. List and sequences of the primers used in this study.
- References (*37*–*40*)

Download PDF

**Other Supplementary Material for this manuscript includes the following:**

- Data file S1 (Microsoft Excel format). List of all gene FPKMs for each patient and control.
- Data file S2 (Microsoft Excel format). Supervised study of DEGs for each group of patients compared to controls.

**Files in this Data Supplement:**

- Adobe PDF - aau9425\_SM.pdf
