# Supplementary material for: Transcriptional profiling at the DLK1/MEG3 domain explains clinical overlap between imprinting disorders
Source: Sci Adv. 2019 Feb 20;5(2):eaau9425. doi: 10.1126/sciadv.aau9425 (PMC6382400; doi:10.1126/sciadv.aau9425)
Supplement: http://advances.sciencemag.org/cgi/content/full/5/2/eaau9425/DC1 [file aau9425_SM.pdf]

Science Advances
AAAS

advances.sciencemag.org/cgi/content/full/5/2/eaau9425/DC1

Supplementary Materials for

Transcriptional profiling at the *DLK1/MEG3* domain explains clinical overlap between imprinting disorders

Walid Abi Habib*, Frédéric Brioude, Salah Azzi, Sylvie Rossignol, Agnès Linglart, Marie-Laure Sobrier, Éloïse Giabicani, Virginie Steunou, Madeleine D. Harbison, Yves Le Bouc, Irène Netchine*

*Corresponding author. Email: walid.abi-habib@inserm.fr (W.A.H.); irene.netchine@aphp.fr (I.N.)

Published 20 February 2019, *Sci. Adv.* **5**, eaau9425 (2019)
DOI: 10.1126/sciadv.aau9425

The PDF file includes:

Supplementary Methods
Fig. S1. Expression of 14q32.2 MEGs and 11p15.5 and 15q11-q13 PEGs in five different passages of cultured fibroblasts from TS14 patients.
Fig. S2. Schematic representation of the subcloned transcripts of MEG8 (*MEG8a* and *MEG8b*).
Fig. S3. Distribution of minor allele frequency according to combined exome and RNA-seq data for fibroblasts from the TS14-1, TS14-2, and SRS/TS14 patients.
Fig. S4. Schematic representation of the four putative imprinted loci identified on the basis of allele-specific gene expression and DMRs.
Fig. S5. Schematic representation of the molecular findings and the hypothesized mechanism from this study.
Table S1. Methylation levels for all patients and controls, as determined by ASMM RT-qPCR.
Table S2. Clinical features for TS14 patients with imprinting defects at the *DLK1/MEG3* domain described here.
Table S3. The allelic status of imprinted genes, as determined by RNA and exome sequencing for TS14 and control fibroblasts.
Table S4. List of all genes with monoallelic expression, as determined by RNA and exome sequencing in TS14 patients.
Table S5. List and sequences of the primers used in this study.
References (*37–40*)

Other Supplementary Material for this manuscript includes the following:

(available at advances.sciencemag.org/cgi/content/full/5/2/eaau9425/DC1)

Data file S1 (Microsoft Excel format). List of all gene FPKMs for each patient and control.

Data file S2 (Microsoft Excel format). Supervised study of DEGs for each group of patients compared to controls.

Supplementary Methods

Skin-derived fibroblast cultures

Skin-derived fibroblasts from patients and controls were cultured to confluence in RPMI 1640 (Gibco, Cergy Pontoise, France) supplemented with 10% fetal calf serum and 50 U/ml ampicillin/50 µg/ml streptomycin at 37°C (number of passages <10). Cells were then treated with trypsin and centrifuged. The cell pellet obtained was washed twice with 1x PBS and used for DNA and micro/large-scale RNA extractions.

Reverse transcription and real-time PCR quantification of micro/mRNAs

We synthesized cDNA from miRNAs isolated from fibroblasts and used it for quantitative PCR with the miScript PCR system (Qiagen, France). We synthesized cDNA from 250 ng of small- RNA eluate from fibroblasts, using the miScript II RT Kit with HiSpec buffer. We then diluted 20 µl of cDNA and analyzed it in triplicate (3 ng cDNA per well) with the miScript SYBR Green PCR Kit, consisting of QuantiTect SYBR Green PCR Master Mix and miScript Universal Primer. We purchased miScript Primer Assays from Qiagen, France. Quantitative PCR was performed on an ABI-7900HT machine (Life Technologies, France) with the miScript cycling program. Hs-miR-16-1 was used to normalize fibroblast miRNA levels. Fibroblast cDNA was synthesized from 1 µg of total large RNA, with the SuperScript II Reverse Transcriptase system (Life Technologies, France). It was diluted to obtain a final volume of 200 µl. Gene expression (3 µl of diluted cDNA per well) was quantified with the Power SYBR Green Kit (Life Technologies, France). The amount of each transcript was normalized against β-actin (housekeeping gene stably expressed in both patients and controls). Expression in the controls was arbitrarily set to 1 and fold-changes (FC) between two groups were calculated as FC= $2^{-\Delta\Delta Ct}$. The RT-qPCR primers are listed in Supplementary Table S5.

For the *MEG3* and *MEG8* overexpression assays, we proceeded differently to quantify the levels of expressions for *MEG3* and *MEG8* since residual plasmids were found in our RNA extractions. Therefore, we measured using a standard curve, the numbers of MEG3 and MEG8 copies in both RNA and cDNA solutions for each transfection culture (MEG3, MEG8 and empty vectors), and assessed the number of expressed *MEG3* and *MEG8* by subtracting the number of copies found in the RNA solution from the number found in the cDNA solution (using equal volumes).

Finally, copy numbers were normalized against β-actin levels.

Bisulfite treatment of DNA

Sodium bisulfite treatment converts all the unmethylated cytosine residues of DNA into uracil residues. Methylated cytosine residues are unaffected. This process thus generates C/T polymorphisms, which can be used to distinguish between the methylated and unmethylated alleles. Genomic DNA (1 µg) was treated with sodium bisulfite, with the EZ DNA Methylation kit (Zymo Research, Orange, CA), according to the manufacturer's instructions. It was then eluted in 50 µl RNase-free H_2O and stored at -20°C.

TaqMan allele-specific methylated multiplex real-time quantitative PCR (ASMM RTQ-PCR) and methylation analysis

The methylation status of the 11p15.5 (ICR1 CBS2), 14q32.2 (IG-DMR CG4 and *MEG3*-DMR CG7), 15q11-q13 (IC DMR), 7q32.2 (*PEG1/MEST* DMR) and 16p13.3 (*ZNF597* DMR) loci was assessed by ASMM RT-qPCR, as previously described(*30*-*33*). The methylation index (MI) at each locus was determined by calculating the ratio of methylated to unmethylated alleles as follows: (amount of methylated allele/sum of methylated and unmethylated alleles) x 100. The ASMM RT-qPCR primer and probe sequences are available upon request.

Expression vectors and plasmid constructs

The pCI-MEG3 and pCI-MEG3b plasmids used for *MEG3* overexpression were a gift from Anne Klibanski(*37*) (Addgene plasmid # 44727 and # 59421). The empty mammalian pCI expression vector (E173A) used as a transfection control was purchased from Promega (Madison, USA).
We cloned both isoforms of the *MEG8* transcript by inserting the corresponding sequences into the expression vector pcDNA3.1/V5-His/TOPO (Invitrogen). The resulting constructs were designated pC-MEG8a (NR_024149.2) and pC-MEG8b.

Cell cultures and transfection assays
GM05757 (control human skin-derived fibroblast) cells were cultured in 24-well plates to ~80% confluence, in RPMI 1640 (Gibco, Cergy Pontoise, France) supplemented with 10% fetal calf serum and 50 U/ml ampicillin/50 µg/ml streptomycin, at 37°C. The cells were then cultured in antibiotic-free medium for 24 hours before transfection. They were transfected with empty, *MEG3*- or *MEG8*-containing expression vectors (100 ng pC-MEG8a/pC-MEG8b and 200 ng pCI-MEG3/pCI-MEG3b), in the presence of 0.75 µl Lipofectamine 3000 and 0.175 µl P3000 reagent (Lipofectamine® 3000 Transfection Reagent, Thermo Fisher, France). Twenty-four hours after transfection, the medium was replaced with fresh medium containing antibiotic, and the cells were collected in TRIzol® Reagent (ThermoFisher, France) for RNA extraction 24 h later.

Whole Exome sequencing
Genomic DNA was obtained by Agilent in–solution enrichment (SureSelect kit CRE, Agilent) with the Agilent biotinylated oligonucleotide probes library (kit CRE – 54 Mb, Agilent), and subjected to paired–end 75 base–read massively parallel sequencing on an Illumina HiSeq4000. Sequence capture, enrichment and elution were performed according to the manufacturer's instructions (SureSelect, Agilent), except for library preparation with the NEBNext® Ultra kit (New England Biolabs®). For library preparation, we fragmented 600 ng of each genomic DNA sample by sonication and purified fragments of 150–200 bp. Paired–end adaptor oligonucleotides from the NEB kit were ligated to repaired, A–tailed fragments, which were then purified and enriched by eight cycles of PCR. We then hybridized 1200 ng of these purified libraries to the SureSelect oligomer probe capture library for 72 h. After hybridization, washing, and elution, the eluted fraction was amplified by nine cycles of PCR, purified and quantified by qPCR, to obtain sufficient DNA template for downstream applications. Each eluted enriched DNA sample was then sequenced on an Illumina HiSeq4000, as paired–end 75–bp reads. Image analysis and base calling were performed with Illumina Real–Time Analysis ([[rta]]), using the default parameters.

Bioinfomatics analysis of sequencing data based on the Illumina pipeline (CASAVA1.8.2).
CASAVA was used to align the results of a sequencing run to a reference genome (hg19), to call the SNPs based on allele calls and read depth, and to detect variants (SNPs & Indels). All targets were sequenced to a depth of more than 20X in all patients screened.
The alignment algorithm used was ELANDv2e (multiseed and gapped alignments). Only the positions included in the bait coordinates were conserved. Genetic variants were annotated with the IntegraGen in–house pipeline.
We annotated genes (RefSeq), and known polymorphisms (dbSNP 132, 1000Genome, EVS) and characterized the mutations (exonic, intronic, silent, nonsense). For each position, exomic frequencies (Homo & HTZ) were determined from all the exomes already sequenced at Integragen, and the exome results provided by 1000G, EVS and HapMap. Variants had to be non–synonymous and absent from dbSNP132, 1000Genome, EVS, and HapMap, with the vast majority of bases scoring Q30 or above, to meet our filtering requirements.

mRNA library construction and sequencing
Libraries were prepared with the TruSeq Stranded mRNA kit, according to the manufacturer's protocol. This protocol involves the following steps: purification of PolyA-containing mRNA molecules from 1 µg

total RNA with poly-T oligo-coated magnetic beads, fragmentation with divalent cations at high temperature to obtain fragments of about 300 bp, double-stranded cDNA synthesis and Illumina adapter ligation and cDNA library amplification by PCR for sequencing. Sequencing was then performed on an Illumina HiSeq4000, with 75 bp paired-end reads.

mRNA sequence alignment and quantification of gene expression
Read quality was assessed for each sample with FastQC (http://www.bioinformatics.babraham.ac.uk/projects/fastqc). A subset of 500,000 reads from each Fastq file was aligned against the reference human genome hg19/GRCh37 with tophat2, to determine insert sizes with Picard. Full Fastq files were aligned against the reference human genome hg19/GRCh37 with tophat2 (-p 24 -r 150 -g 2 --library-type fr-firststrand)(*38*). We removed reads mapping to multiple locations. We used HTSeq to obtain the number of reads associated with each gene in the Gencode v19 database, restricting the analysis to protein-coding genes, antisense and lincRNAs (n=32601). We used the Bioconductor DESeq package(*39*) to import raw HTSeq counts for each sample into R statistical software, and to extract the count matrix. After normalization for library size, we normalized the count matrix for gene coding sequence length, to calculate compute FPKM scores (number of fragments per kilobase of exon model and millions of mapped reads). Bigwig visualization files were generated with the bam2wig python script(*40*).

Unsupervised mRNA-seq analysis
We imported FPKM values into R statistical software. The $\log_2$ normalized expression matrix from the 500 most variant genes (based on the standard deviation) was used to classify the samples according to their gene expression patterns, by principal component analysis (PCA), hierarchical clustering and consensus clustering. We used standard R functions for the PCA and hierarchical clustering (with Euclidean distance and the Ward method). We used consensus clustering (Bioconductor ConsensusClusterPlus package) to assess the stability of the clusters. We established consensus partitions of the dataset into K clusters (for K = 2, 3, ..., 8), on the basis of 1,000 resampling iterations (80% of genes, 80% of samples) of hierarchical clustering, with Pearson's dissimilarity as the distance metric and Ward's method for linkage analysis. We used the cumulative distribution functions (CDFs) of the consensus matrices to determine the optimal number of clusters (K = 3), considering both the shape of the functions and the area under the CDF curves.

Supervised mRNA-seq analysis
We used the Bioconductor DESeq package3 to import raw HTSeq counts into R statistical software, to obtain size factors and dispersion estimates, and to test for differential expression. We applied a FPKM≥1 and q-value threshold of ≤0.05 to define differentially expressed genes and we used hypergeometric tests to identify gene sets from the MSigDB v5 (*41*) database that were overrepresented among the lists of over- or underexpressed genes, with correction for multiple testing according to the Benjamini-Hochberg procedure.

Detection of variants in exome data
Raw sequence alignment and variant calling were performed with Illumina CASAVA 1.8 software. CASAVA aligns reads to the human reference genome (hg19), using the alignment algorithm ELANDv2, and then calls single-nucleotide variants (SNVs) on the basis of allele calls and read depth. We considered only positions located within genes and we applied stringent quality controls to ensure the retention of reliable positions with two different alleles only: sequencing depth ≥10 reads, with ≥5 variant calls representing 20% to 70% of total reads, and QPHRED score ≥30 for both SNP detection and genotype calling.

Allelic expression in mRNAseq data
We used the bamreadcount(*42*) tool to assess base counts in the bam files at each informative genomic

position (i.e. positions with two different nucleotides). We filtered out positions with a coverage of less than 10. For each position, we calculated the minor allele frequency: base counts for the minor allele over total base counts. Genes for which all SNPs were informative, with a minor allele frequency of at least 0.1, were confidently classified as biallelically expressed, whereas genes for which all SNPs were informative, with a minor allele frequency below 0.1 were considered to be monoallelically expressed. Data on all of the monoallelically expressed genes listed in supplementary table S4 were taken from http://suprahex.r-forge.r-project.org/RNAseq_geneinfo.txt.

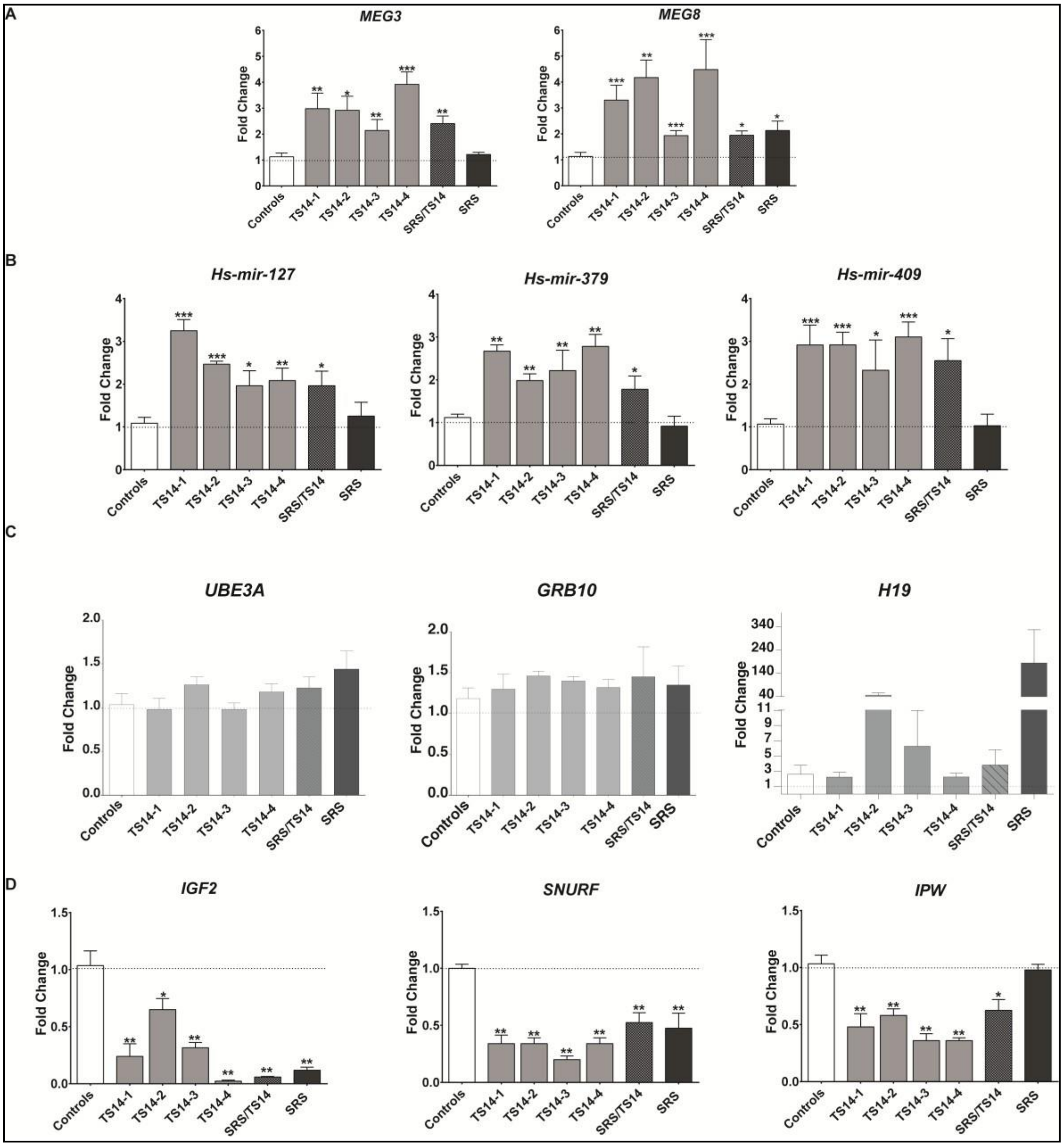

Fig. S1. Expression of 14q32.2 MEGs and 11p15.5 and 15q11-q13 PEGs in five different passages of cultured fibroblasts from TS14 patients. The data shown are mean values for **(A)** the 14q32.2 lncRNAs *MEG3* and *MEG8*, **(B)** three miRNAs from the 14q32.2 domain, **(C)** three MEGs and **(D)** the 11p15.5 and 15q11-q13 PEGs ± SEM for 5 different passages for TS14 and SRS/TS14 skin-derived fibroblast cultures and a pool of five donors for controls and SRS patients.

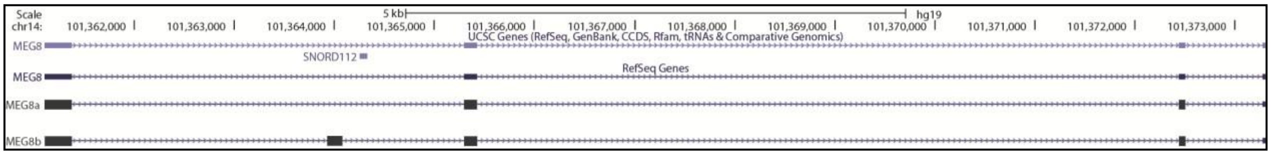

Fig. S2. Schematic representation of the subcloned transcripts of MEG8 (*MEG8a* and *MEG8b*). Comparison with the reference sequence. *MEG8a* corresponds to NR_024149.2, whereas *MEG8b* has an extra exon located between exons 1 and 2.

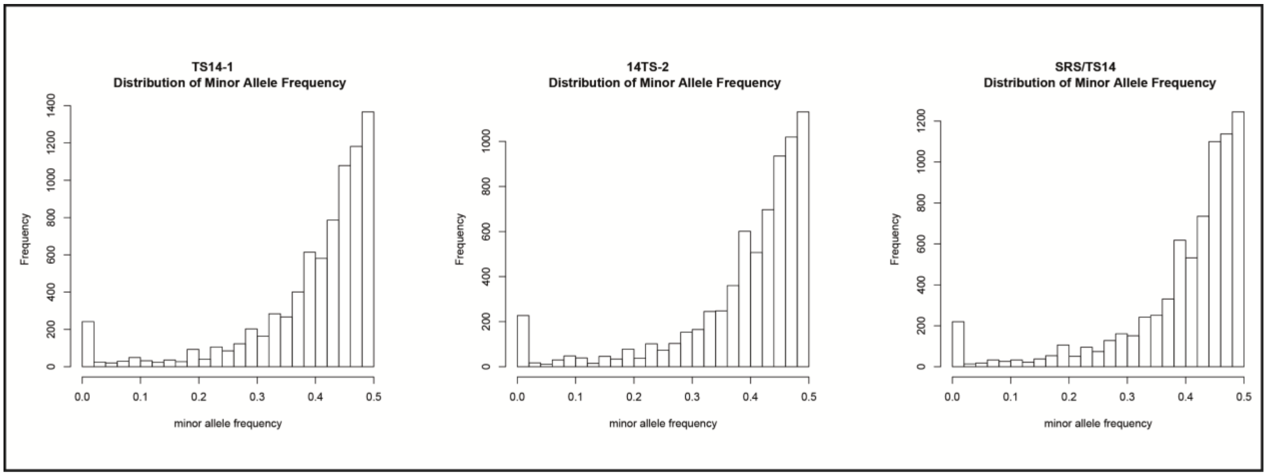

Fig. S3. Distribution of minor allele frequency according to combined exome and RNA-seq data for fibroblasts from the TS14-1, TS14-2, and SRS/TS14 patients.

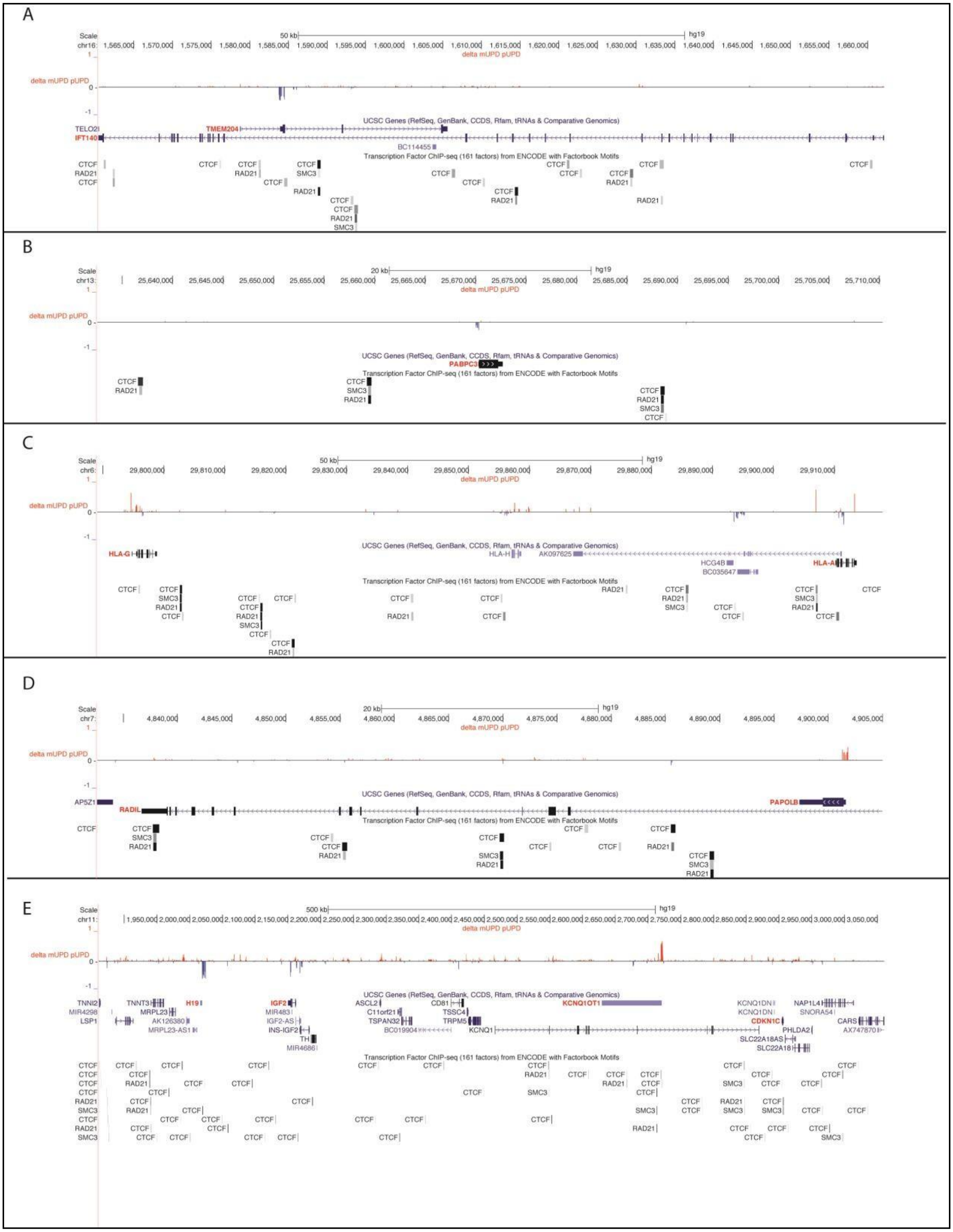

Fig. S4. Schematic representation of the four putative imprinted loci identified on the basis of allele-specific gene expression and DMRs. For determination of the imprinting status of these genes, we checked for differentially methylated regions in the gene promoters, and in a 500 kb window, using methylation data from whole-genome unimaternal and unipaternal blood cell-derived DNA (*43*) and data for hemimethylation and the presence of CTCF/cohesion binding sites from the ENCODE project. We identified four new clusters, and seven putative imprinted genes. Delta mUPD pUPD is the difference

in methylation at the same site in the DNA between maternal uniparental disomies "mUPD" paternal uniparental disomies "pUPD". Red and blue bars represent maternally and paternally expressed DMRs respectively. **(A)** The first cluster is located on chromosome region 16p13.3, with the *TMEM204* gene displaying monoallelic expression and a DMR for paternal expression in the promoter. The neighboring gene, *IFT140*, has also been reported to display monoallelic expression (*44*). In addition, the *TMEM204* promoter and the gene body harbor several CTCF and cohesion binding sites, which are often found around imprinted genes. **(B)** The second putative imprinted gene is *PABPC3*, located on chromosome region 13q12.13, which also has a paternal DMR in its promoter. The two other loci harbored two putative imprinted genes each: *HLA*-A/*HLA*-G **(C)** and *PAPOLB*/*RADIL* **(D)**, located in chromosome regions 6p22.1 and 7p22.1 respectively, both with maternal DMRs and CTCF/cohesion binding sites. **(E)** Example of the imprinted loci in the 11p15.5 region, showing the maternal DMR in the *KCNQ1OT1/CDKN1C* domain and the paternal DMR in the *IGF2/H19* domain.

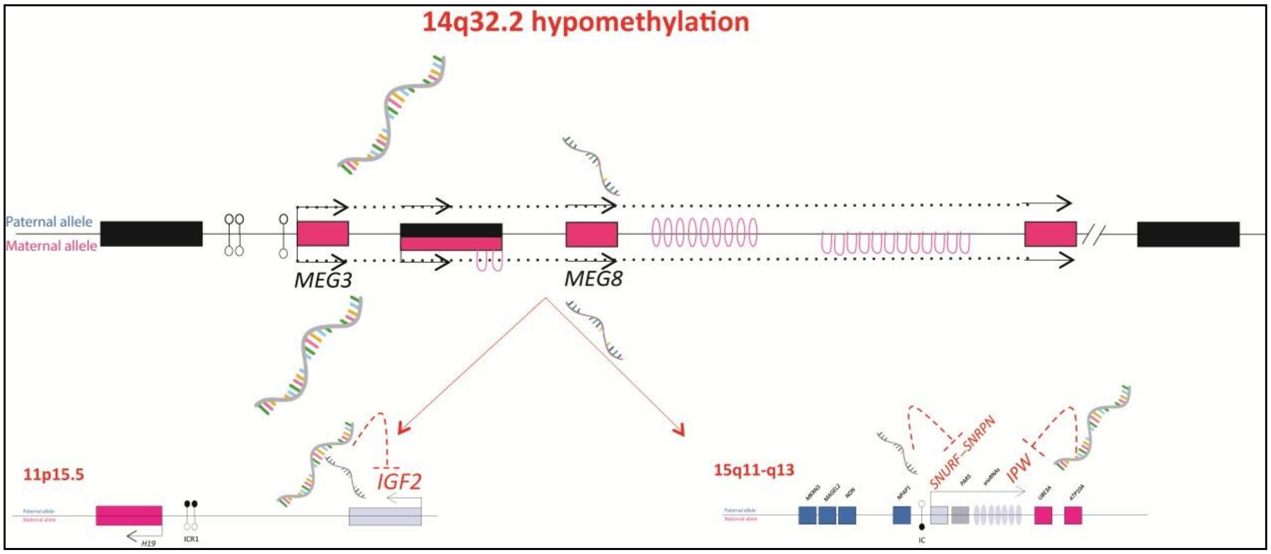

Fig. S5. Schematic representation of the molecular findings and the hypothesized mechanism from this study. Hypomethylation of 14q32.2 imprinted *DLK1/MEG3* domain affects the expression not only of genes within this locus, but also of other imprinted genes on chromosomes 11 and 15. We propose a mechanism in which *MEG3* and *MEG8* regulate in *trans* the expressions of *IGF2* (synergic action), *IPW* and SNURF (in an independent manner).

Table S1. Methylation levels for all patients and controls, as determined by ASMM RT-qPCR.

Fibroblasts extracted DNA

TS14 Patients	ICR1 CBS2	H19DMR	IG-DMR	MEG3-DMR	PLAGL1	PEG/MEST	GRB10	IC-SNRPN	ICR2	ZNF597-DMR
TS14-1	46	48	0	0	59	39	49	46	49	50
TS14-2	49	50	3	0	52	38	51	46	48	51
TS14-3	50	50	10	0	55	48	49	45	49	49
TS14-4	51	48	3	0	51	47	48	47	42	52
SRS/TS14	12	14	2	0	48	39	49	48	50	29

SRS Patients	ICR1 CBS2	H19DMR	IG-DMR	MEG3-DMR	PLAGL1	PEG/MEST	GRB10	IC-SNRPN	ICR2	ZNF597-DMR
SRS 1	6	0	50	50	51	52	48	48	45	50
SRS 2	7	0	50	48	56	48	52	52	49	49
SRS 3	0	0	48	45	48	48	50	50	49	51
SRS 4	3	0	47	48	56	41	45	45	48	51
SRS 5	0	0	45	49	55	48	49	49	45	48

Controls	ICR1 CBS2	H19DMR	IG-DMR	MEG3-DMR	PLAGL1	PEG/MEST	GRB10	IC-SNRPN	ICR2	ZNF597-DMR
C1	55	50	46	50	51	46	48	52	50	51
C2	48	45	41	48	54	47	50	42	46	47
C3	52	48	45	46	54	46	49	53	48	48
C4	58	50	51	48	50	50	51	49	49	49
C5	41	40	44	50	49	49	49	45	50	52

Leukocytes extracted DNA

TS14 Patients	ICR1 CBS2	H19DMR	IG-DMR	MEG3-DMR	PLAGL1	PEG/MEST	GRB10	IC-SNRPN	ICR2	ZNF597-DMR
TS14-1	55	53	7	0	50	53	52	45	52	45
TS14-2	54	50	0	0	51	52	47	54	47	52
TS14-5	51	52	5	3	50	52	48	47	50	43
TS14-6	50	50	21	23	48	46	50	45	50	50
TS14-7	48	51	26	26	53	51	47	45	50	54
TS14-8	50	49	7	0	49	53	50	48	51	49
TS14-9	50	51	0	0	49	50	53	49	52	51

Table S2. Clinical features for TS14 patients with imprinting defects at the *DLK1*/*MEG3* domain described here.

Patient	TS14-1	TS14-2	TS14-3	TS14-4	SRS/TS14	TS14-5	TS14-6	TS14-7	TS14-8	TS14-9
**Available biological material**	Fibroblasts/Serum	Fibroblasts	Fibroblasts	Fibroblast	Fibroblasts	Serum	Serum	Serum	Serum	Serum
**Age (years)**	3.4	5.6	13.4	12	13.2	9.5	4.2	17.7	18.3	4.5
**Sex**	F	F	F	F	F	F	M	F	F	F
**SRS NH-CSS**	5/6	3/5	4/5	3/5	6/6	3/6	5/6	4/6	5/6	5/6
**SGA (birth weight &/or length)**	YES	YES	YES	YES	YES	YES	YES	YES	YES	YES
**Postnatal growth failure**	YES	YES	YES	YES	YES	NO	YES	NO	NO	NO
**Relative macrocephaly at birth**	NO	NO	ND	ND	YES	NO	NO	NO	YES	YES
**Protruding/prominent forehead**	YES	YES	YES	YES	YES	YES	YES	YES	YES	YES
**Body asymmetry**	NO	NO	YES	NO	YES	NO	YES	YES	YES	YES
**Early feeding difficulties and/or BMI<-2SDS**	YES	NO	NO	NO	YES	YES	YES	YES	YES	YES
**Hypotonia**	YES	YES	YES	ND	YES	NO	NO	ND	ND	YES
**Down-turned corners of the mouth**	YES	YES	YES	YES	YES	YES	YES	YES	NO	NO
**Lowset or posteriorly rotated ears**	YES	YES	YES	YES	YES	YES	NO	YES	YES	NO
**Diagnosed cognitive disabilities or motor delay**	YES	YES	YES	NO	NO	NO	NO	NO	NO	NO
**Age at start of marked increase in BMI (fat mass)**	ND	ND	4	6.5	3.1	1	ND	3.3	1	ND
**Small hands**	NO	ND	YES	YES	YES	YES	NO	YES	YES	NO
**Small feet**	NO	ND	YES	YES	YES	YES	NO	YES	YES	NO
**Clinodactyly**	NO	YES	YES	YES	YES	YES	YES	NO	YES	NO
**Early puberty**	NA	NA	YES	YES	YES	YES	NA	YES	YES	NA
**Pubertal onset**	NA	NA	4	8	6.5	6.6	NA	8.5	9.5	NA

Table S3. The allelic status of imprinted genes, as determined by RNA and exome sequencing for TS14 and control fibroblasts.

Monoallelic expression (MAE)			
	Gene (#SNV)	Imprinting status	Imprinting status in fibroblasts (Santoni 2017)
TS14-1	H19 (9)	Imprinted	NA
	PRIM2 (16)	Conflictingdata	MAE
TS14-2	OSBPL5 (1)	Imprinted	MAE
	NDN (1)	Imprinted	NA
	ZDBF2 (1)	Imprinted	MAE
	PEG10 (1)	Imprinted	MAE
	PRIM2 (17)	Conflictingdata	MAE

Biallelic expression (BAE)			
	Gene (#SNV)	Imprinting status	Imprinting status in fibroblasts (Santoni 2017)
TS14-1	SLC22A18(3)	Imprinted	BAE
	PHLDA2 (1)	Imprinted	NA
	RB1 (1)	Imprinted	BAE
	ATP10A (1)	Imprinted	BAE
	DNMT1 (3)	Imprinted	BAE
	DGCR6L (1)	Imprinted	NA
	CPA4 (2)	Imprinted	MAE
	ZFAT (3)	Imprinted	BAE
	ZFAT-AS1(1)	Imprinted	BAE
TS14-2	SLC22A18(3)	Imprinted	BAE
	ZC3H12C (2)	Imprinted	BAE
	NTM (1)	Imprinted	BAE
	ATP10A (6)	Imprinted	BAE
	DNMT1 (1)	Imprinted	BAE
	AIM1 (4)	Imprinted	BAE
	GRB10 (2)	Imprinted	BAE
	SGCE (1)	Imprinted	BAE
	CPA4 (2)	Imprinted	MAE
	ZFAT (5)	Imprinted	BAE
	ZFAT-AS1(1)	Imprinted	BAE
SRS/TS14	H19 (1)	Imprinted	NA
	SLC22A18(1)	Imprinted	BAE
	ATP10A (4)	Imprinted	BAE
	ZNF597 (1)	Imprinted	MAE
	DNMT1 (1)	Imprinted	BAE
	AIM1 (4)	Imprinted	BAE
	CPA4 (2)	Imprinted	MAE

NA: Not assessed MAE: Mono-Allelicly Expressed BAE: Bi-Allelicly Expressed

Table S4. List of all genes with monoallelic expression, as determined by RNA and exome sequencing in TS14 patients.

Gene	Description	Gene_biotype	Gene_position	TS14-1	TS14-2	SRS/TS14	n/3
OSTF1	osteoclast stimulating factor 1	protein_coding	chr9:77703459-77762181	MAE			1
TEK	TEK tyrosine kinase, endothelial	protein_coding	chr9:27109139-27230173		MAE		1
COL15A1	collagen, type XV, alpha 1	protein_coding	chr9:101705461-101833069		MAE		1
NIPAL2	NIPA-like domain containing 2	protein_coding	chr8:99202061-99306621	MAE			1
PCMTD1	protein-L-isoaspartate (D-aspartate) O-methyltransferase domain containing 1	protein_coding	chr8:52730140-52811735			MAE	1
AC084125.4		antisense	chr8:145660602-145665354	MAE			1
TONSL		protein_coding	chr8:145654165-145669827	MAE			1
EPPK1	epiplakin 1	protein_coding	chr8:144939497-144952632	MAE			1
PABPC1	poly(A) binding protein, cytoplasmic 1	protein_coding	chr8:101698044-101735037			MAE	1
KIAA1324L	KIAA1324-like	protein_coding	chr7:86506222-86689015		MAE		1
PAPOLB	poly(A) polymerase beta (testis specific)	protein_coding	chr7:4897364-4901625		MAE		1
RADIL	Rap Associating With DIL Domain)	protein_coding	chr7:4797055-4883719		MAE		1
EEPD1	endonuclease/exonuclease/phosphatase family domain containing 1	protein_coding	chr7:36192758-36341152		MAE		1
INTS1	integrator complex subunit 1	protein_coding	chr7:1509913-1545489	MAE			1
AGAP3	ArfGAP with GTPase domain, ankyrin repeat and PH domain 3	protein_coding	chr7:150782918-150841523	MAE			1
KIAA1147	KIAA1147	protein_coding	chr7:141356528-141401953	MAE			1
CHCHD3	coiled-coil-helix-coiled-coil-helix domain containing 3	protein_coding	chr7:132469629-132766848			MAE	1
METTL2B	methyltransferase like 2B	protein_coding	chr7:128116783-128146656		MAE		1
NAA38	N(alpha)-acetyltransferase 38, NatC auxiliary subunit	protein_coding	chr7:117824086-117832878			MAE	1
NAMPT	nicotinamide phosphoribosyltransferase	protein_coding	chr7:105888731-105926772	MAE	MAE	MAE	3
HLA-A	major histocompatibility complex, class I, A	protein_coding	chr6:29909037-29913661		MAE	MAE	2
HLA-G	major histocompatibility complex, class I, G	protein_coding	chr6:29794744-29798902	MAE			1
DUSP22	dual specificity phosphatase 22	protein_coding	chr6:291630-351355			MAE	1
BTN2A2	butyrophilin, subfamily 2, member A2	protein_coding	chr6:26383324-26395102		MAE		1
TPMT	thiopurine S-methyltransferase	protein_coding	chr6:18128542-18155305	MAE	MAE		2
MLLT4	myeloid/lymphoid or mixed-lineage leukemia (trithorax homolog, Drosophila); translocated to, 4	protein_coding	chr6:168227602-168372703		MAE		1
MRPL18	mitochondrial ribosomal protein L18	protein_coding	chr6:160210844-160219468		MAE		1
BCLAF1	BCL2-associated transcription factor 1	protein_coding	chr6:136578001-136610989	MAE	MAE	MAE	3
ERAP2	endoplasmic reticulum aminopeptidase 2	protein_coding	chr5:96211643-96255420	MAE		MAE	2
HNRNPH1	heterogeneous nuclear ribonucleoprotein H1 (H)	protein_coding	chr5:179041179-179061785		MAE		1
KIF4B	kinesin family member 4B	protein_coding	chr5:154393260-154397685		MAE		1
G3BP1	GTPase activating protein (SH3 domain) binding protein 1	protein_coding	chr5:151150606-151192346	MAE	MAE		2
C5orf46	chromosome 5 open reading frame 46	protein_coding	chr5:147260289-147286101		MAE		1
SRA1	steroid receptor RNA activator 1	protein_coding	chr5:139916925-139937895			MAE	1
ZNF608	zinc finger protein 608	protein_coding	chr5:123972608-124084500		MAE		1
SEC31A	SEC31 homolog A (S. cerevisiae)	protein_coding	chr4:83739814-83822319	MAE			1
PDGFRA	platelet-derived growth factor receptor, alpha polypeptide	protein_coding	chr4:55095264-55164414		MAE		1
FIP1L1	factor interacting with PAPOLA and CPSF1	protein_coding	chr4:54243810-55161439		MAE		1
RPL9	ribosomal protein L9	protein_coding	chr4:39455744-39460568		MAE	MAE	2
NEIL3	nei endonuclease VIII-like 3 (E. coli)	protein_coding	chr4:178230990-178284097	MAE			1
MED28	mediator complex subunit 28	protein_coding	chr4:17616254-17635728	MAE			1
SFRP2	secreted frizzled-related protein 2	protein_coding	chr4:154701744-154710272	MAE			1
ZNF717	zinc finger protein 717	protein_coding	chr3:75758794-75834734		MAE		1
TMF1	TATA element modulatory factor 1	protein_coding	chr3:69068978-69101484			MAE	1
CTD-2013N24.2		antisense	chr3:69063092-69105773			MAE	1
PXK	PX domain containing serine/threonine kinase	protein_coding	chr3:58318607-58411748	MAE			1
RPSA	ribosomal protein SA	protein_coding	chr3:39448180-39454033		MAE		1
PLD1	phospholipase D1, phosphatidylcholine-specific	protein_coding	chr3:171318195-171528740		MAE		1
PFN2	profilin 2	protein_coding	chr3:149682691-149768575	MAE			1
GATA2	GATA binding protein 2	protein_coding	chr3:128198270-128212028		MAE		1
KALRN	kalirin, RhoGEF kinase	protein_coding	chr3:123798870-124445172		MAE		1
ITGB2	integrin, beta 2 (complement component 3 receptor 3 and 4 subunit)	protein_coding	chr21:46305868-46351904		MAE		1
THNSL2	threonine synthase-like 2 (S. cerevisiae)	protein_coding	chr2:88469835-88486146	MAE			1
TET3	tet methylcytosine dioxygenase 3	protein_coding	chr2:74229840-74335303	MAE			1
C1D	C1D nuclear receptor corepressor	protein_coding	chr2:68268262-68338080	MAE			1
STK36	serine/threonine kinase 36	protein_coding	chr2:219536749-219567439		MAE		1
ARPC2	actin related protein 2/3 complex, subunit 2, 34kDa	protein_coding	chr2:219081817-219119079			MAE	1
PRKRA	protein kinase, interferon-inducible double stranded RNA dependent activator	protein_coding	chr2:179296141-179316239		MAE		1
RBMS1	RNA binding motif, single stranded interacting protein 1	protein_coding	chr2:161128662-161350305	MAE	MAE		2
RPS28	ribosomal protein S28	protein_coding	chr19:8386042-8388224		MAE		1
CERS4	ceramide synthase 4	protein_coding	chr19:8271620-8327305	MAE			1
ZNF587	zinc finger protein 587	protein_coding	chr19:58361225-58376480	MAE	MAE		2
ZNF667-AS1	ZNF667 antisense RNA 1 (head to head)	lincRNA	chr19:56988619-57012035		MAE		1
ZNF677	zinc finger protein 677	protein_coding	chr19:53731577-53758151			MAE	1
PTOV1	prostate tumor overexpressed 1	protein_coding	chr19:50353992-50364001			MAE	1
CCDC61	coiled-coil domain containing 61	protein_coding	chr19:46498339-46524576	MAE			1
OPA3	optic atrophy 3 (autosomal recessive, with chorea and spastic paraplegia)	protein_coding	chr19:46030685-46105470			MAE	1
PSG4	pregnancy specific beta-1-glycoprotein 4	protein_coding	chr19:43696854-43711451			MAE	1
C19orf55	chromosome 19 open reading frame 55	protein_coding	chr19:36249044-36261930			MAE	1
CEP89	centrosomal protein 89kDa	protein_coding	chr19:33369902-33462897		MAE		1
TLE2	transducin-like enhancer of split 2 (E(sp1) homolog, Drosophila)	protein_coding	chr19:2997636-3047633			MAE	1
ZNF100	zinc finger protein 100	protein_coding	chr19:21905568-21950430			MAE	1
CTC-260E6.6		antisense	chr19:20236186-20432114			MAE	1
ZNF93	zinc finger protein 93	protein_coding	chr19:20011722-20046384	MAE			1
SSBP4	single stranded DNA binding protein 4	protein_coding	chr19:18529674-18545372	MAE			1
LPHN1	latrophilin 1	protein_coding	chr19:14260750-14316999		MAE		1
CTB-55O6.12		antisense	chr19:14247991-14282079		MAE		1
ZNF700	zinc finger protein 700	protein_coding	chr19:12035883-12061588		MAE		1
PRKCSH	protein kinase C substrate 80K-H	protein_coding	chr19:11546109-11561783			MAE	1
TIMM21	translocase of inner mitochondrial membrane 21 homolog (yeast)	protein_coding	chr18:71815746-71826197	MAE			1
DSG2	desmoglein 2	protein_coding	chr18:29078006-29128971	MAE			1
ROCK1	Rho-associated, coiled-coil containing protein kinase 1	protein_coding	chr18:18526867-18691812	MAE			1
USP14	ubiquitin specific peptidase 14 (tRNA-guanine transglycosylase)	protein_coding	chr18:158383-214629		MAE	MAE	2
ACADVL	acyl-CoA dehydrogenase, very long chain	protein_coding	chr17:7120444-7128592		MAE	MAE	2
CDC27	cell division cycle 27	protein_coding	chr17:45195069-45266788		MAE	MAE	2
COPRS	coordinator of PRMT5, differentiation stimulator	protein_coding	chr17:30178883-30186356		MAE		1
MAP2K3	mitogen-activated protein kinase kinase 3	protein_coding	chr17:21187984-21218552	MAE			1
HS3ST3B1	heparan sulfate (glucosamine) 3-O-sulfotransferase 3B1	protein_coding	chr17:14204400-14252721		MAE		1
HS3ST3A1	heparan sulfate (glucosamine) 3-O-sulfotransferase 3A1	protein_coding	chr17:13399006-13505244		MAE	MAE	2
NPIPB15	nuclear pore complex interacting protein family, member B15	protein_coding	chr16:74411776-74425978		MAE		1
CES1	carboxylesterase 1	protein_coding	chr16:55836763-55867249			MAE	1
ZNF598	zinc finger protein 598	protein_coding	chr16:2047655-2059824			MAE	1
RPS2	ribosomal protein S2	protein_coding	chr16:2012053-2014861		MAE	MAE	2
TMEM204	transmembrane protein 204	protein_coding	chr16:1578689-1605581			MAE	1
GABARAPL3	GABA(A) receptors associated protein like 3, pseudogene	protein_coding	chr15:90890819-90892669	MAE			1
CSPG4	chondroitin sulfate proteoglycan 4	protein_coding	chr15:75966663-76005189		MAE		1
NUSAP1	nucleolar and spindle associated protein 1	protein_coding	chr15:41624892-41673248	MAE			1
HERC2	HECT and RLD domain containing E3 ubiquitin protein ligase 2	protein_coding	chr15:28356186-28567298	MAE			1
SNORD116-20	small nucleolar RNA, C/D box 116-20	lincRNA	chr15:25332808-25334043			MAE	1
ACOT2	acyl-CoA thioesterase 2	protein_coding	chr14:74034324-74042357			MAE	1

L3HYPDH	L-3-hydroxyproline dehydratase (trans-)	protein_coding	chr14:59927081-59951148		MAE		1
RP11-290D2.6		antisense	chr13:45914174-45915318			MAE	1
TPT1	tumor protein, translationally-controlled 1	protein_coding	chr13:45911008-45915505			MAE	1
CSNK1A1L	casein kinase 1, alpha 1-like	protein_coding	chr13:37677398-37679803	MAE		MAE	2
PABPC3	poly(A) binding protein, cytoplasmic 3	protein_coding	chr13:25670300-25673389			MAE	1
PARP4	poly (ADP-ribose) polymerase family, member 4	protein_coding	chr13:24995064-25086948		MAE		1
SLC2A3	solute carrier family 2 (facilitated glucose transporter), member 3	protein_coding	chr12:8071826-8088871		MAE		1
NAV3	neuron navigator 3	protein_coding	chr12:78224685-78606790	MAE			1
MLF2	myeloid leukemia factor 2	protein_coding	chr12:6857170-6876641			MAE	1
RP11-386G11.10		antisense	chr12:49521565-49541652	MAE			1
TUBA1B	tubulin, alpha 1b	protein_coding	chr12:49521565-49525180	MAE			1
PARP11	poly (ADP-ribose) polymerase family, member 11	protein_coding	chr12:3900213-3982608		MAE		1
OVCH1-AS1	OVCH1 antisense RNA 1	protein_coding	chr12:29542227-29640421		MAE		1
FAM101A	family with sequence similarity 101, member A	protein_coding	chr12:124457788-124800570		MAE		1
ZNF664	zinc finger protein 664	protein_coding	chr12:124456392-124499986		MAE		1
EIF2S3L	Putative eukaryotic translation initiation factor 2 subunit 3-like protein	protein_coding	chr12:10658201-10675734	MAE	MAE		2
SCYL2	SCY1-like 2 (S. cerevisiae)	protein_coding	chr12:100660918-100735502	MAE			1
NRIP3	nuclear receptor interacting protein 3	protein_coding	chr11:9002123-9025596			MAE	1
RPL27A	ribosomal protein L27a	protein_coding	chr11:8703958-8736306		MAE		1
SAA2	serum amyloid A2	protein_coding	chr11:18260770-18270190	MAE	MAE		2
EI24	etoposide induced 2.4	protein_coding	chr11:125439112-125454575	MAE	MAE	MAE	3
CSNK2A3	casein kinase 2, alpha 3 polypeptide	protein_coding	chr11:11373489-11374904		MAE		1
TM9SF3	transmembrane 9 superfamily member 3	protein_coding	chr10:98277866-98347209	MAE	MAE		2
SYT15	synaptotagmin XV	protein_coding	chr10:46955444-46971400		MAE		1
RP11-38L15.3		antisense	chr10:46951472-46966835		MAE		1
NAMPTL	nicotinamide phosphoribosyltransferase-like	protein_coding	chr10:36810649-36813162	MAE		MAE	2
FANK1	fibronectin type III and ankyrin repeat domains 1	protein_coding	chr10:127585108-127698161	MAE	MAE	MAE	3
ECHDC3	enoyl CoA hydratase domain containing 3	protein_coding	chr10:11784365-11806069		MAE		1
USMG5	up-regulated during skeletal muscle growth 5 homolog (mouse)	protein_coding	chr10:105148798-105156223	MAE			1
AK4	adenylate kinase 4	protein_coding	chr1:65613232-65697828	MAE			1
COL16A1	collagen, type XVI, alpha 1	protein_coding	chr1:32117848-32169920		MAE		1
SLC35E2B	solute carrier family 35, member E2B	protein_coding	chr1:1592939-1624167		MAE		1
FLG	filaggrin	protein_coding	chr1:152274651-152297679		MAE		1
FLG-AS1	FLG antisense RNA 1	antisense	chr1:152140601-152339163		MAE		1
ANP32E	acidic (leucine-rich) nuclear phosphoprotein 32 family, member E	protein_coding	chr1:150190717-150208504	MAE			1
NOTCH2NL	notch 2 N-terminal like	protein_coding	chr1:145209119-145291972	MAE	MAE		2
GSTM4	glutathione S-transferase mu 4	protein_coding	chr1:110198703-110208118		MAE		1
C1orf159	chromosome 1 open reading frame 159	protein_coding	chr1:1017198-1051741			MAE	1

Table S5. List and sequences of the primers used in this study.

Primers used for mRNA quantification by RT-PCR	
Primer	3' sequence 5'
MEG3 F	AGAACTGCGGATGGAAGCTG
MEG3 R	CCCACATTCGAGGTCCCTTC
MEG8 F	CAGCCTTCCAGACTCGCTTG
MEG8 R	TCTTCTAGAGCCCCAGATCCC
H19 F	GGTGGCCATGAAGATGGAGT
H19 R	GTGTGACGGCGAGGACAG
HOTS F	AGAGATAGCGACACGTGGGT
HOTS R	GGACCTCACTCCTTCCCCAT
IGF2 F	TGGACACCCTCCAGTTCGTC
IGF2 R	GCGGAAACAGCACTCCTCAA
SNURF F	GTCTGAGGAGCGGTCAGT
SNURF R	GGCAGTCCTTCTGCGTTTGA
IPW F	TGCCTAGACCACCCACTAAAGG
IPW R	AGTCTCCATGCGGAAGGAAGA
β-Actin F	AGAGCTACGAGCTGCCTGAC
β-Actin R	AGCACTGTGTTGGCGTACAG

Primers used for gDNA and cDNA amplification and sequencing	
MEG3 g/msF	GCCATCACCTGGATGCCTAC
MEG3 g/msR	TTTTCATTGTCAAATGTCAGAGGC
H19-exon1-F	CAGTCACCCGGCCCAGAT
H19-exon1-R	AAGACACCATCGGAACAGCA

Primers used for MEG8 cDNA amplification, sequencing and cloning	
MEG8-cF	GGTCTGAAAAATGATATTCATTGTCC
MEG8-cR	CGAGCAAGTCACTAACCTCTCCA
